# A Novel Method Based on Combination of Independent Component Analysis and Ensemble Empirical Mode Decomposition for Removing Electrooculogram Artifacts From Multichannel Electroencephalogram Signals

**DOI:** 10.3389/fnins.2021.729403

**Published:** 2021-10-11

**Authors:** Chao-Lin Teng, Yi-Yang Zhang, Wei Wang, Yuan-Yuan Luo, Gang Wang, Jin Xu

**Affiliations:** ^1^The Key Laboratory of Biomedical Information Engineering of Ministry of Education, School of Life Science and Technology, Xi’an Jiaotong University, Xi’an, China; ^2^The Key Laboratory of Neuro-informatics and Rehabilitation Engineering of Ministry of Civil Affairs, Xi’an, China; ^3^National Engineering Research Center for Healthcare Devices, Guangzhou, China; ^4^Department of Psychiatry, The First Affiliated Hospital, Xi’an Jiaotong University, Xi’an, China; ^5^Department of Psychology, Xi’an Mental Health Center, Xi’an, China

**Keywords:** electrooculogram (EOG), artifacts, electroencephalogram (EEG), ensemble empirical mode decomposition (EEMD), independent component analysis (ICA)

## Abstract

Electrooculogram (EOG) is one of common artifacts in recorded electroencephalogram (EEG) signals. Many existing methods including independent component analysis (ICA) and wavelet transform were applied to eliminate EOG artifacts but ignored the possible impact of the nature of EEG signal. Therefore, the removal of EOG artifacts still faces a major challenge in EEG research. In this paper, the ensemble empirical mode decomposition (EEMD) and ICA algorithms were combined to propose a novel EEMD-based ICA method (EICA) for removing EOG artifacts from multichannel EEG signals. First, the ICA method was used to decompose original EEG signals into multiple independent components (ICs), and the EOG-related ICs were automatically identified through the kurtosis method. Then, by performing the EEMD algorithm on EOG-related ICs, the intrinsic mode functions (IMFs) linked to EOG were discriminated and eliminated. Finally, artifact-free IMFs were projected to obtain the ICs without EOG artifacts, and the clean EEG signals were ultimately reconstructed by the inversion of ICA. Both EOGs correction from simulated EEG signals and real EEG data were studied, which verified that the proposed method could achieve an improved performance in EOG artifacts rejection. By comparing with other existing approaches, the EICA obtained the optimal performance with the highest increase in signal-to-noise ratio and decrease in root mean square error and correlation coefficient after EOG artifacts removal, which demonstrated that the proposed method could more effectively eliminate blink artifacts from multichannel EEG signals with less error influence. This study provided a novel promising method to eliminate EOG artifacts with high performance, which is of great importance for EEG signals processing and analysis.

## Introduction

Electroencephalogram (EEG) can be measured from electrodes placed on the human scalp and directly reflects electrical activity linked to the central nervous system. As a convenient non-invasive neuroimaging technology, EEG has been extensively applied to the diagnosis or prediction of mental disorders (Rojas et al., 2018; Wang et al., 2020), the evaluation of drug actions (Cornelissen et al., 2015; Li et al., 2020), patient health monitoring (Sivathamboo et al., 2019), the research of artificial intelligence (Gandhi et al., 2014) and rehabilitation engineering (Kranczioch et al., 2014), and so on. Unfortunately, recorded EEG signals may be severely contaminated by power line interferences and different types of artifacts, such as electrooculogram (EOG), electromyogram (EMG), and electrocardiogram (ECG). Among these artifact signals, EOG produced by eye blinks is the most common artifact. The amplitude of EOG signal is much higher than that of EEG. Besides, the energy of EOG is mainly concentrated in the low frequency band, which overlaps the EEG basic rhythm waves. EOG artifacts could lead to incorrect results and bias in subsequent EEG analysis. Therefore, the removal of EOG artifacts is a very essential and important preprocess step for EEG signals.

A well-known adaptive filtering (ADF) approach based on regression is applied to remove blink artifacts (He et al., 2004; Wallstrom et al., 2004). It is assumed that the measured EEG signals are composed by mixing true EEG and EOG linearly. Once the proportion of EOG in the recorded EEG is estimated, the EOG signals multiplied by the proportion are subtracted from the raw EEG signals to obtain corrected EEG data. But in fact, this method does not take into account a cross-contamination between EEG and EOG signals. Another most classic method based on the independent component analysis (ICA) algorithm has been widely employed for artifacts rejection in EEG signals. Many researches showed that EOG-related and EOG-free independent components (ICs) could be successfully separated from the contaminated EEG signals by using ICA methods, respectively (Iriarte et al., 2003; Flexer et al., 2005; Li et al., 2006). Thus, ICs associated with EOG artifacts could be removed or replaced with zeros by manual or automatic ways according to EEG waveforms, typical morphology, topographical map, the methods of artifact detection, etc. On the one hand, manual intervention to reject the artifactual ICs is a tedious and time-consuming process; on the other hand, because artifact components still contain some brain electrical activity of interest, a straightforward elimination of these artifact components implies the considerable loss of some important EEG data. Besides, other blind source separation (BSS) techniques, such as second-order blind identification (SOBI), stationary subspace analysis (SSA), and canonical correlation analysis (CCA), have also been used for artifacts removal (Ng and Raveendran, 2009; Zeng et al., 2013; Janani et al., 2018). Moreover, wavelet transform is also a common artifact removal technique that relies on predefined mother wavelet and decomposition level (Krishnaveni et al., 2006; Rajesh et al., 2012). After decomposing signal into approximate and detail coefficients, an appropriate threshold is then used on the coefficients to identify and eliminate artifacts. However, the choices of the mother wavelet and the threshold are empirical, especially for the threshold, which have a great influence on the denoising result.

Additionally, because of its data-driven, adaptive, and no prior knowledge, empirical mode decomposition (EMD) is considered as an ideal tool for non-stationary EEG signals analysis and process (Huang et al., 1998). Recently, the EMD method was successfully applied in the field of the suppression of blink artifacts (Rashed-Al-Mahfuz et al., 2013). However, the EMD method is highly sensitive to noise and faces a problem of the mode mixing, which will make extracted intrinsic mode functions (IMFs) inaccurate. To overcome this dilemma, the noise-assisted version of the EMD algorithm called ensemble EMD (EEMD) was proposed by Wu and Huang (2009). Nevertheless, both EMD and EEMD approaches directly remove artifact-linked IMFs while losing much useful cerebral activities.

To further improve the performance of artifacts removal, many attempts have been made to propose a new technique for artifacts rejection based on the above methods. Castellanos and Makarov presented a novel wavelet-enhanced ICA methodology (wICA) to eliminate artifacts in EEG signals by implementing a wavelet thresholding on the decomposed ICs not the original signals as an intermediate process (Castellanos and Makarov, 2006). In addition, Mammone et al. (2012) developed an approach based on discrete wavelet transform (DWT) and ICA, termed as AWICA, to automatically detect and reject artifacts. For EOG artifacts suppression, the AWICA method outperformed the wICA technique. Subsequently, another new technique was introduced by Klados et al. (2011) for rejecting the artifacts of eye blink by combining blind source separation and regression-based adaptive filtering. The experimental results revealed that the proposed methodology could successfully remove EOG artifacts. Zhao et al. (2014) combined DWT and an adaptive predictor filter for blink artifacts rejection from the raw EEG signals. For simulated and collected data, the proposed method was proved that it was capable of removing EOG artifacts without using EOG signal. In recent years, Mowla et al. (2015) reported artifacts elimination using a new technique combined of SOBI and stationary wavelet transform (SWT) (SOBI-SWT). Comparative results showed that the proposed method was more effective than other techniques. Wang et al. (2016) combined ICA and multivariate empirical mode decomposition (MEMD) to propose a new automatic EOG artifacts removal method (IMEMD), whose advantages were tested by simulated EEG data with comparisons of several methods. Very recently, by a joint use of EEMD and CCA, a novel EEMD-CCA approach was proposed by Chen et al. (2019) for removing artifacts. Generally, it was demonstrated that those different combined methods can evidently enhance the performance of artifacts removal. However, there still existed drawbacks of those methods. For instance, the selection of optimal mother wavelets for wavelet transform is practically difficult, and the cross-contamination between EEG and EOG affects the performance of regression-based methods for artifacts removal.

In this study, by combining the EEMD and ICA algorithms, we proposed a novel EEMD-based ICA (EICA) method to remove blink artifacts from multichannel contaminated EEG signals. Both simulation and real EOG artifacts correction were studied to verify the effectiveness of the new approach. Moreover, some existing artifact removal approaches were employed in comparison with the proposed method.

The paper is organized as follows: section “Materials and Methods” briefly described experimental data including simulated and actual EEG data and then introduced the methodologies presented in this study; in section “Results” of EOG artifacts rejection using the proposed method was presented, and other artifact removal algorithms were used for comparison; and section “Discussion” of results and a summary conclusion was drawn in the final section.

## Materials and Methods

### Electroencephalogram Data

Two different types of EEG datasets were used to test the validity of the proposed artifact rejection algorithm in this study. The first dataset was simulated EEG data with EOG artifacts produced by mixing real EOG with pure EEG signals. The second dataset was real EEG data acquired from 10 healthy subjects, which was contaminated by blink artifacts. The description of two EEG datasets was shown as follows.

#### Generation of Simulated Data

**FIGURE 1:**
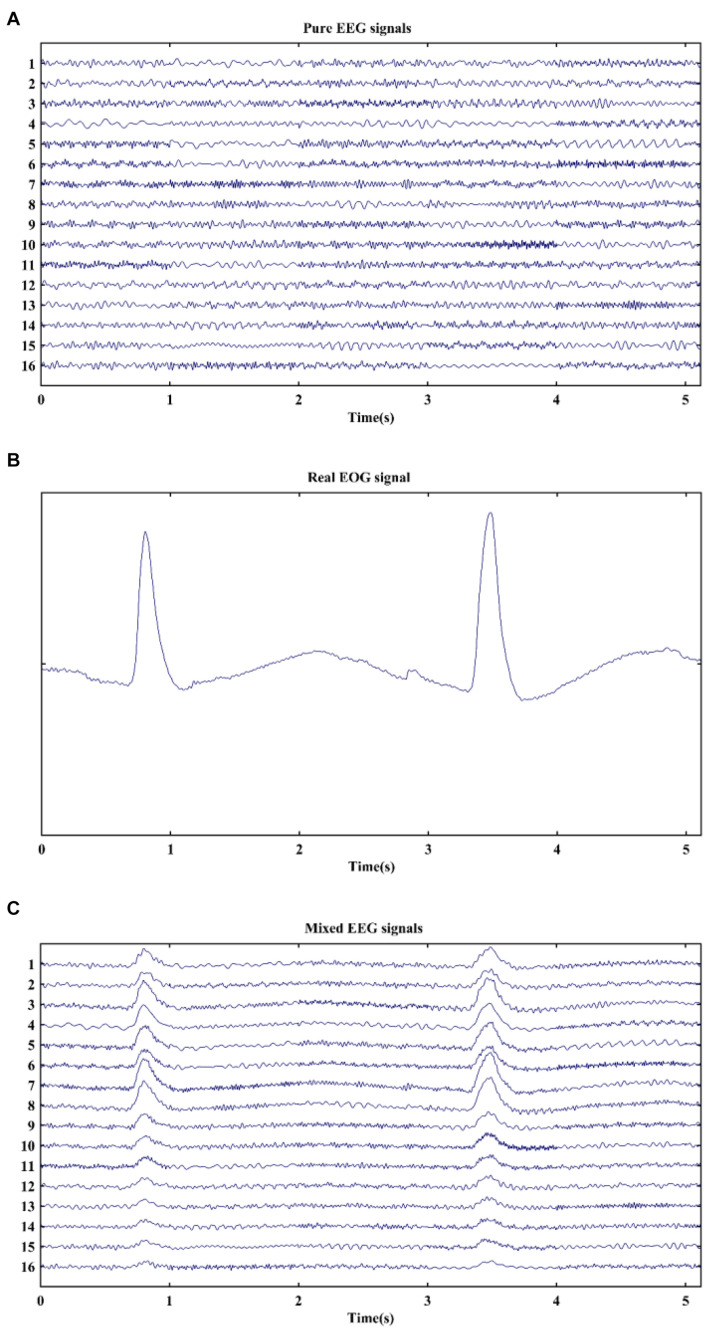
Typical examples of signals in simulation. **(A)** Pure EEG signals, **(B)** real EOG signal, and **(C)** mixed EEG signals.

Simulated EEG data with a sampling frequency of 1,000 Hz was artificially generated by MATLAB function developed by the authors of Yeung et al. (2007). The noise.m function was employed to produce pure EEG signals, which were constructed by summing four phase- and frequency-randomized sinusoids. The phases were chosen randomly varying between 0 and 2π, and the frequencies spanned the range from 0.5 to 35 Hz. In our simulation, 160 datasets of 16-channel pure EEG signals were generated, which were denoted by the matrix *A* (size, 16 × 5,120). A segment of real EOG signal was selected from the EOG recordings with a sampling rate of 1,000 Hz and a band-pass filter of 0.5–5 Hz, which was denoted by the matrix *B* (size, 1 × 5,120). Simulated EEG signals and a real EOG signal are shown in Figures 1A,B. The artificially EOG-contaminated EEG signals were then created by mixing matrix *B* into matrix *A* with different weighted amplitude for each channel, which were expressed in the following formula (Gao et al., 2010):

(1) $$C\,\left(i,:\right)=A\,\left(i,:\right)+\lambda_{i}*B,i\in \left\{1,2,\ldots ,16\right\}$$

where matrix *C* and λ_*i*_ are the mixed EEG signals with EOG artifacts and the adjusted parameter, respectively. By changing the value of λ_*i*_, signal-to-noise ratio (SNR) values were set from −15 to 0 dB with a 1 dB step to mimic distribution of eye blinks on the scalp. Thus, each SNR value corresponded to one EEG channel. An example of simulated mixed signals is displayed in Figure 1C. It is clearly observed that the pure EEG signals are severely contaminated by EOG artifacts at 0.6–1.1 s and 3.2–3.7 s. Finally, 160 datasets of EEG signals with EOG artifacts were generated for each SNR in the simulation.

#### Acquisition of Real Electroencephalogram Data

The real EEG signals were collected from 32 scalp electrodes using the Neuroscan EEG acquisition system (Neuroscan Inc., Charlotte, NC, United States). The positions of all electrodes (Ag/AgCl) conformed to the standard international 10–20 system. Two linked electrodes on the left and right mastoids were used as reference, and a ground electrode was placed on the forehead. In addition, the horizontal electrooculogram (HEOG) and vertical electrooculogram (VEOG) were recorded using two pairs of bipolar electrodes in both horizontal and vertical directions. Electrode impedance was maintained below 5 kΩ. EEG and EOG were recorded continuously at a sampling rate of 1,000 Hz under emotional faces recognition task. Stimuli contained target stimuli (presented by positive and negative faces) and non-target stimuli (presented by neutral faces) with a probability of 0.5 and 0.5, respectively. When stimuli were presented on a standard LCD monitor with a black background, subjects were instructed to respond to target stimuli as accurately as possible and ignore non-target stimuli. While responding to target stimuli, subjects were asked to remain quiet at the same time and only move their right index finger to press a button. Besides, we also recorded the signals under resting state with eyes open. In this study, our main purpose was to verify that the proposed method could successfully remove blink artifacts from contaminated EEG signals. Therefore, 18 channels of EEG with eye blinks were picked out for artifacts elimination study: Fp1, Fp2, F7, F3, Fz, F4, F8, FT7, FC3, FCz, FC4, FT8, C3, C4, P3, P4, O1, and O2. The data are preprocessed by a 0.5–35 Hz band-pass filter. Ten healthy subjects (male/female = 4/6; mean age, 25.80 ± 5.71 years) were involved in the study. An average of 23.3 EEG segments (*SD* = 1.6) were obtained from resting state and 23.1 segments (*SD* = 1.6) from task state. The length of EEG segments ranged from 2 to 8 s. All participants were in good physical condition and given to sign the informed written consent. The study was reviewed and approved by the local ethics committee of Xi’an Jiaotong University.

### Methodology

**FIGURE 2:**
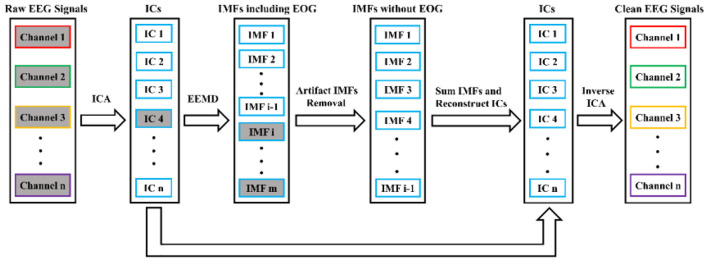
Algorithm block diagram for EOG artifacts removal. The rectangles filled with gray denote artifact-linked components, and the IC4 is decomposed by EEMD.

In the study, ICA and EEMD were combined to use for EOG artifacts correction. The proposed EOG artifacts removal algorithm flowchart is shown in Figure 2. There were five steps included. First, multichannel EEG signals were separated into multiple ICs by means of ICA method. Second, EOG-related ICs were automatically identified by the kurtosis value, while the rest of ICs were retained. Third, the EEMD method was applied to decompose EOG-related ICs into a set of IMFs. Fourth, IMFs linked to EOG artifacts were automatically recognized by correlation coefficient and eliminated. Finally, the clean EEG signals without blink artifacts were ultimately reconstructed by projecting IMFs and using the inverse transform of ICA sequentially.

#### Independent Component Analysis

As one kind of common BSS techniques, ICA is extensively used in artifacts detection and removal from EEG signals. The basic idea underlying the ICA method is to extract statistically independent sources called ICs from the observed signals using higher-order statistic (Comon, 1994). The aim of ICA attempts to determine a demixing matrix *W* and estimate unknown independent source signals *s*(*t*) = [*s*_1_(*t*),*s*_2_(*t*),,*s*_*n*_(*t*)]*^T^*. Thus, the ICA problem can be expressed by the following equation: *s*(*t*) = *wx*(*t*). In this study, *x*(*t*) = [*x*_1_(*t*),*x*_2_(*t*),,*x*_*n*_(*t*)]*^T^* denotes *n* channels raw EEG signals containing EOG artifacts. Since EOG and EEG are produced by different independent sources, EOG can be separated from EEG signals. The original signals were then decomposed by the ICA algorithm to acquire multiple ICs. In this study, the information-maximization algorithm of ICA was employed (Bell and Sejnowski, 1995).

#### Electrooculogram-Related Independent Components Identification

After EOG-contaminated EEG signals were decomposed into EOG-related ICs *s*_*EOG*_(*t*) = [*s*_1_(*t*),*s*_2_(*t*),,*s*_*m*_(*t*)]*^T^* and artifact-free ICs *s*_*EEG*_(*t*) = [*s*_*m* + 1_(*t*),*s*_*m* + 2_(*t*),,*s*_*n*_(*t*)]*^T^*, ICs linked to EOG artifacts needed to be distinguished. The simple threshold method may have difficulty to detect artifacts generated by small eye blinks. However, high-order statistics of signals can reflect more information about EOG artifacts that low-order statistics does not have. The fourth-order cumulant, termed as kurtosis, was regarded as the evaluation criterion for differentiating the EOG-related ICs (Delorme et al., 2007; Mammone et al., 2012). Moreover, kurtosis is a measure of signal peaks that has simple computation and theory. Given that *x* is a scalar random variable, the kurtosis can be represented as the following expression:

(2) $$k=m_{4}-3\,m_{2}^{2}$$

where *m*_*n*_ = *E*{(*x*−*m*_1_)*^n^*}is the *n*th order central moment of the variable, and *m*_1_ is the mean value of random variable. Generally, kurtosis is zero for a Gaussian signal and negative for “flat” activity distributions (such as sub-Gaussian). On the other hand, kurtosis is positive for super-Gaussian distributions with “peak” activities, typical artifacts such as ECG and EOG (Delorme et al., 2007; Mammone et al., 2012). Thus, ICs related to blink artifacts could be discriminated by measuring kurtosis value for each component. The selection of the practical threshold is determined by experimental data, which is the result of trials and errors. In this study, the critical threshold value was set at 1.5 (Mammone et al., 2012; Wang et al., 2016). Then, the ICs whose kurtosis values exceeded the threshold value were automatically specified as EOG-related components. According to the above-mentioned artifactual components, each IC in *s*_*EOG*_(*t*) was served as the input of EEMD analysis and further was separated into multiple IMFs including cerebral activity and eye blink artifacts.

#### Ensemble Empirical Mode Decomposition

The EMD method, first pioneered in 1998 by Huang et al. (1998) can adaptively decompose a multicomponent signal into a finite set of band-limited intrinsic oscillatory modes called IMFs by the local characteristic time scales of the signal. For EMD algorithm, each IMF should satisfy two properties: (1) in the whole data set, the number of local extrema and the number of zero crossings must be equal or differ at most by one, and (2) the mean value of envelops defined by local maxima and minima is zero at any point. In addition, the frequency bands of these IMF components vary from high to low frequencies. While EEMD is an extension of EMD method, it not only has advantages of the EMD but also solves the mixing mode problem. Thus, EEMD has been widely applied to the processing of electrophysiological signals (Chen et al., 2010; Hassan and Bhuiyan, 2017). For EEMD algorithm, it defines the IMF components by averaging the ensemble of the corresponding IMFs obtained by employing EMD to the analyzed signal with the addition of independent, identically distributed white noise of the same standard deviation (Wu and Huang, 2009). Referring to previous research (Chen et al., 2019), the ensemble number (i.e., trials of running EMD) was set to 10 in this work. In other words, the EEMD algorithm repeatedly decomposes the raw signal with added white noise by the EMD algorithm in many trials, which results in an ensemble of IMFs. Finally, the IMFs are achieved by the mean of corresponding IMF sets. Let *s*_*i*_(*t*)(*i* = 1,2,,*m*) be a single channel signal. The EEMD algorithm can be presented as follows:

1. Generate the data by adding a white noise series to the targeted signal: $x_{i}^{h}\,\left(t\right)=s_{i}\,\left(t\right)+w^{h}\,\left(t\right)$, where *w^h^*(*t*) denotes the white noise series.
2. Decompose the data $x_{i}^{h}\,\left(t\right)$ into IMFs by using EMD: $x_{i}^{h}\left(t\right)=\sum_{j=1}^{K}d_{i,j}^{h}\left(t\right)\left(j=1,2,\ldots ,K\right)$, where *j* denotes the mode.
3. The IMFs can be obtained by averaging the corresponding modes: $I\,M\,F_{j}^{i}\,\left(t\right)=1/p\,\sum_{h=1}^{p}d_{i,j}^{h}\,\left(t\right)$, where $I\,M\,F_{j}^{i}\,\left(t\right)$ represents the *j*th mode of *s*_*i*_(*t*), and *p* is the ensemble number.

Since the added white noise is different in each trial, it would be encountered by averaging the ensemble of IMFs. Thus, the signal *s*_*i*_(*t*) can be decomposed as $s_{i}\left(t\right)=\sum_{j=1}^{K}IMF_{j}^{i}\left(t\right)\left(i=1,2,\ldots ,m;j=1.2,\ldots ,K\right)$. Therefore, the original signal can be considered as the sum of all mode components ranked according to their local characteristics in time–frequency domain.

#### Electrooculogram-Related Intrinsic Mode Functions Identification and Rejection

In order to eliminate the EOG-related IMFs, the correlation coefficients between IMFs and EOG signal were used to distinguish EOG-related IMFs with a high degree of correlation between them. Here, given $z_{i}\,\left(t\right)={\left[\sum_{j=1}^{K}I\,M\,F_{j}^{i}\,\left(t\right),\sum_{j=2}^{K}I\,M\,F_{j}^{i}\,\left(t\right),\ldots ,\sum_{j=K}^{K}I\,M\,F_{j}^{i}\,\left(t\right)\right]}^{T}$ is a *K*-channel signal, which is reconstructed by various combinations of IMFs from *s*_*i*_(*t*)(*i* = 1,2,…,*m*). Then, the correlation coefficients between each channel signal of *z*_*i*_(*t*) and EOG were calculated. It is worth noting that the energy of EOG artifacts mainly focuses in low frequency. Since the frequency bands of IMFs were ordered from high to low according to characteristic of signal oscillators, correlation coefficients initially increased and then gradually decreased. As a result, the largest correlation coefficient could be determined as a critical value. If the *p*th correlation coefficient qualified the maximum value, the *p*th IMF to the last IMF were marked as EOG artifacts, which was defined as $u_{i}\,\left(t\right)=\sum_{j=p}^{K}I\,M\,F_{j}^{i}\,\left(t\right)$. Meanwhile, the remaining IMFs without EOG artifacts were reserved, which were presented as $v_{i}\,\left(t\right)=\sum_{j=1}^{p-1}I\,M\,F_{j}^{i}\,\left(t\right)$. Afterward, IMFs linked to EOG were removed by setting all their values to zero.

#### Signal Reconstruction

The signal reconstruction process involved two parts: IMFs projection and ICA inverse transform. The first transform was to transform IMFs to obtain EOG-free ICs *s*_*EOG*−*free*_(*t*) = [*v*_1_(*t*),*v*_2_(*t*),…,*v*_*m*_(*t*)]*^T^*. Subsequently, artifact-free ICs *s*_*EOG*−*free*_(*t*) and undecomposed ICs *s*_*EEG*_(*t*) were all projected into the original space to obtain clean EEG signals.

### Quantitative Measures

The performance of EOG artifacts elimination was quantitatively evaluated by three most popular ways, i.e., signal-noise-ratio (SNR), root mean square error (RMSE), and cross-correlation coefficient (CRC). SNR and RMSE were employed for study based on simulated EEG data, while CRC was for study based on real EEG data. Moreover, the objective assessment criteria were also applied for performance comparisons between the proposed algorithm and other existing artifact rejection techniques.

The value of SNR shows the ratio of signal to noise. The larger the SNR value is, the more the noise rejected in the mixed signal is. Here, SNR of EEG is employed to quantify the degree of the removal of blink artifact from EEG signal, which is given as $S\,N\,R=10*\text{lg}\,\left(\sum_{i=1}^{n}x^{2}\,\left(i\right)/\sum_{i=1}^{n}{\left(y\,\left(i\right)-x\,\left(i\right)\right)}^{2}\right)$, where *x*(*i*) is the pure EEG signal, *y*(*i*) represents the clean EEG after removing EOG artifact, and *n* is the total number of sample points.

The RMSE is defined as $R\,M\,S\,E=\sqrt{1/n\,\sum_{i=1}^{n}{\left(y\,\left(i\right)-x\,\left(i\right)\right)}^{2}}$, where *x*(*i*) denotes the clear EEG signal without EOG artifact, *y*(*i*) represents the reconstructed EEG signal after the elimination of blink artifact, and *n* is the total number of sample points in each channel signal. The RMSE can evaluate the preserved degree of useful EEG information in the reconstructed EEG signal after EOG artifact removal. Therefore, the smaller the RMSE value is, the more useful EEG data will be retained.

The CRC between two signals can be calculated by $C\,R\,C=\frac{C\,o\,v\,\left(X,Y\right)}{\sqrt{V\,a\,r\,\left(X\right)*V\,a\,r\,\left(Y\right)}}$, where *X* and *Y* represent two types of signals, *Cov* and *Var* denote covariance and variance, respectively. The absolute value of CRC is used in this study, and it ranges from 0 to 1. The large values denote two signals with high degree of linear correlation. Specially, CRC = 1 indicates linear correlation between two signals. Therefore, the CRC between removed artifact of eye blink and EOG could be used to determine whether EOG artifact is removed or not.

### Statistical Analysis

The statistical analysis was performed on the MATLAB platform. If two groups of data satisfied normal distribution checked by Kolmogorov–Smirnov test, performance comparisons were analyzed using independent sample *t*-test, otherwise using Mann–Whitney *U*-test. The value of *p* < 0.05 (two sided) was considered to be statistically significant. In addition, false discovery rate (FDR) was used for multiple comparison correction.

## Results

### Eliminating Electrooculogram Artifacts From Simulated Signals

One hundred sixty datasets of EOG-contaminated EEG signals at each SNR value were involved in the study. SNR and RMSE were both used to evaluate the denoising performance of algorithms. In order to find the advantage and disadvantage of the proposed method, other artifact removal algorithms (i.e., ADF, Inf-ICA, CCA, SSA, AWICA, IMEMD, EEMD-CCA, and SOBI-SWT) were employed in this comparison task. In addition, the method based on the combination of EEMD and ICA (EEMD-ICA), first introduced by Mijovic et al. (2010) for source extraction in single-channel EEG signal, was also compared against the proposed method in case of SNRs with −15, −8, and 0 dB. These competitor algorithms are presented in Table 1.

**TABLE 1:** The artifact removal algorithms employed for comparison with the proposed method.

Algorithm	Abbreviation	Article
Adaptive filtering approach based on regression	ADF	He et al., 2004; Wallstrom et al., 2004
Independent component analysis based on information-maximization algorithm	Inf-ICA	Bell and Sejnowski, 1995
Canonical correlation analysis	CCA	De Clercq et al., 2006
Stationary subspace analysis	SSA	Zeng et al., 2013
Automatic wavelet independent component analysis	AWICA	Mammone et al., 2012
Combination of independent component analysis and multivariate empirical mode decomposition	IMEMD	Wang et al., 2016
An approach based on ensemble empirical mode decomposition and canonical correlation analysis	EEMD-CCA	Chen et al., 2019
By combining second-order blind identification and stationary wavelet transform	SOBI-SWT	Mowla et al., 2015
Combination of ensemble empirical mode decomposition and independent component analysis	EEMD-ICA	Mijovic et al., 2010

**FIGURE 3:**
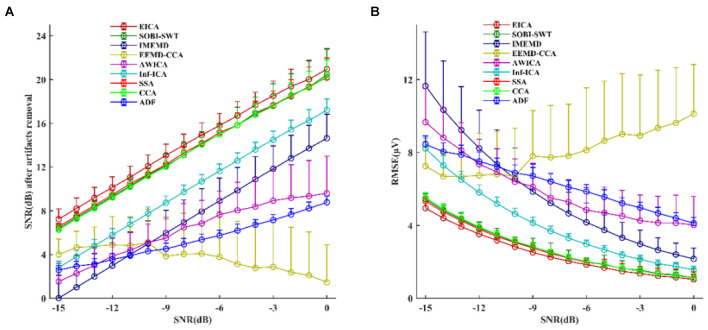
The simulated study: the performance comparisons between the proposed method and other compared methods after removing EOG artifacts from mixed EEG signals in terms of **(A)** SNR and **(B)** RMSE. The circles and error bars represented the mean and standard deviation of SNR in **(A)** and RMSE in **(B)** for 160 denoising simulated EEG datasets.

An example of EOG artifacts removal from mixed EEG signals by different methods is shown in Supplementary Figure 1. By observing the corrected signals, it could be clearly seen that EICA, SOBI-SWT, Inf-ICA, SSA, and CCA effectively eliminated EOG artifacts compared to the other methods (Supplementary Figures 1C,E,M,O,Q). Although EOG artifacts were also removed by EEMD-CCA and ADF, the reconstructed EEG signals showed serious distortion of waveforms (Supplementary Figures 1I,S). It was also obvious that both IMEMD and AWICA methods did not thoroughly remove EOG artifacts for the first few channels (Supplementary Figures 1G,K). In addition, Supplementary Figure 1 shows the extracted signals obtained by subtracting the corrected EEG from pure EEG signals, and we found that the lost EEG information of the proposed EICA method was less than that of other methods. The comparative results of SNRs and RMSEs after suppressing EOG artifacts are presented in Figure 3. The mean and standard deviation values of SNRs and RMSEs were computed for 160 denoising simulated EEG datasets. Apparently, the performance of EICA, SOBI-SWT, SSA, and CCA methods was much better than the other approaches at each SNR condition in terms of SNRs in Figure 3A and RMSEs Figure 3B after EOG artifacts rejection. The proposed algorithm performed the best in the whole SNR range among all the algorithms. Furthermore, the IMEMD method had the worst performance in the low SNR values while the EEMD-CCA approach performed the worst in the high SNR values. Therefore, we further investigated whether the performance of the four algorithms (EICA, SOBI-SWT, SSA, and CCA) had a statistically significant difference. For the whole SNR range, the statistical results showed that SNR values obtained by EICA were significantly higher than that by SOBI-SWT (*p* < 0.05 at SNR = 0 dB, *p* < 0.01 at other SNR values), and the EICA method achieved much lower RMSE values than SOBI-SWT (*p* < 0.05 at SNR = 0 dB, *p* < 0.01 at other SNR values). The SNR and RMSE performance of the EICA method had also statistically significant differences for all SNR values compared to that of the other approaches (*p* < 0.01). Therefore, the simulation results indicated that the proposed method rejected EOG artifacts from contaminated EEG more effectively than the other methods. The results also indicated that SOBI-SWT performed better than CCA under each SNR condition with regard to the performance of SNR and RMSE, and the differences were statistically significant (*p* < 0.01). Besides, when SNR was less than −9 dB, SSA had a better performace of SNR and RMSE than CCA (*p* < 0.01).

Additionally, Table 2 shows the SNR and RMSE results after EOG artifacts removal by the EEMD-ICA and EICA methods. For each case of SNR, the performance of EICA outperformed EEMD-ICA.

**TABLE 2:** Performance of SNR(dB) and RMSE(μV) values after EOG artifacts removal using EEMD-ICA and the proposed method.

SNR(dB)	Algorithm	
	EEMD-ICA	Proposed
−15	−6.48 ± 2.83	7.24 ± 0.93
	25.33 ± 8.82	4.95 ± 0.51
−8	−1.54 ± 2.14	14.02 ± 1.00
	13.90 ± 3.36	2.26 ± 0.27
0	4.57 ± 2.38	20.96 ± 1.88
	6.93 ± 1.88	1.04 ± 0.26

*For each SNR, the upper and lower rows correspond to SNR and RMSE values after EOG artifacts removal, respectively.*

### Electrooculogram Artifacts Rejection of Real Electroencephalogram Data

**FIGURE 4:**
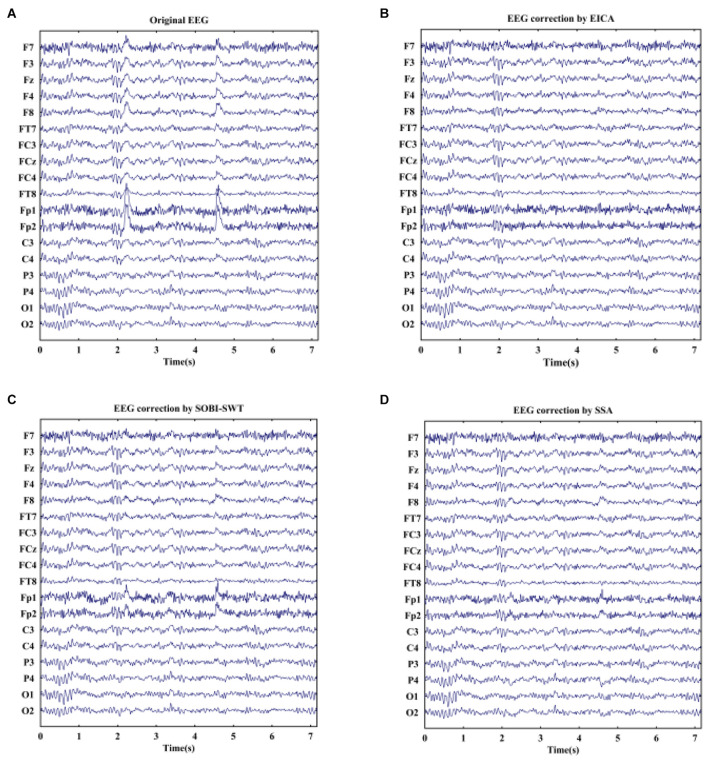
**(A)** A segment of real EEG signals with obvious blink artifacts under eyes-open state and the reconstructed EEG after artifacts removal by using **(B)** EICA, **(C)** SOBI-SWT, and **(D)** SSA.

**FIGURE 5:**
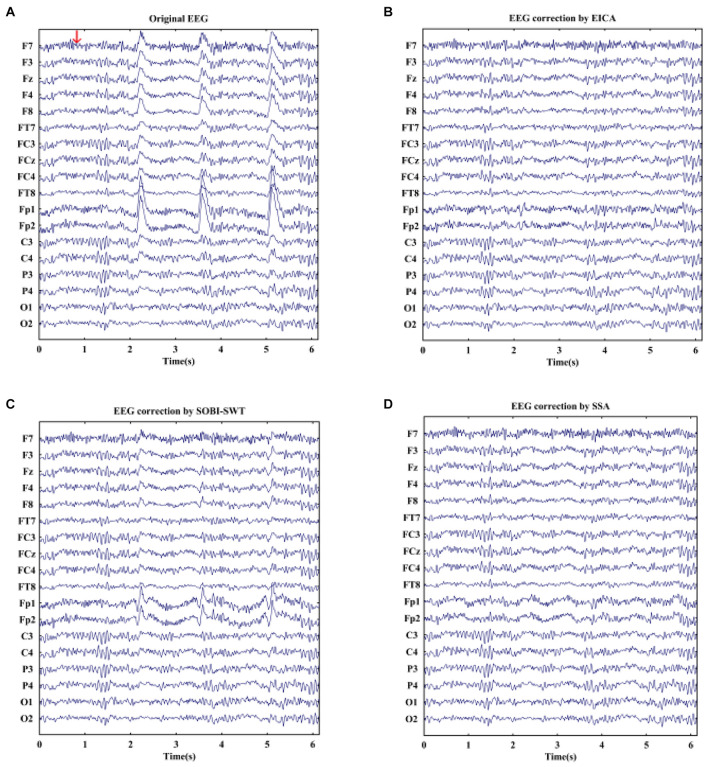
**(A)** A segment of EOG-contaminated EEG signals during emotional faces recognition task and the reconstructed EEG after artifacts removal by using **(B)** EICA, **(C)** SOBI-SWT, and **(D)** SSA. The arrow in **(A)** denotes the appearance of a stimulus.

EOG artifacts rejection from real EEG signals could further verify the effectiveness of the proposed algorithm. Furthermore, the SOBI-SWT and SSA methods were employed to these data as benchmark. Figure 4 displays an example of raw real EEG signals with eye blinks under resting state and the corrected EEG signals by the EICA, SOBI-SWT, and SSA methods. As shown in Figure 4A, a segment of EEG was contaminated by eye blinks before eliminating EOG artifacts. Note that blink artifacts with large amplitude were obviously visible in frontal channels. Figures 4B–D show the clean EEG signals after performing artifacts removal methods. With a comparison of the EEG waves, it could be clearly seen that the EICA and SSA methods removed EOG artifacts more thoroughly than the SOBI-SWT method. Apparently, the corrected EEG signals by SOBI-SWT contained EOG components, especially in prefrontal region. During emotional faces recognition task, a segment of original EOG-contaminated EEG signals is demonstrated in Figures 5A,B–D show the denoising waveforms by using the approaches. From Figures 5B,D, artifacts of eye blink were successfully eliminated by the proposed method and SSA. However, the corrected EEG signals by means of SOBI-SWT still contained residual eye activity in Figure 5C, which was mainly presented in Fp1 and Fp2. To check the waveforms carefully, we can observe in Figures 4B,D that the proposed EICA approach preserved more EEG details than SSA, such as channels Fp1, Fp2, and F7. Besides, although the EOG artifacts were eliminated by SSA, there still existed artifact components contaminating some channels in Figure 5D, such as Fp1 and Fp2.

The average CRC values between removed EOG artifacts and EOG reference for 10 subjects are shown in Table 3. The higher the CRC value is, the more thoroughly the artifact of eye blink is removed. It can be seen that the CRC values by using the proposed approach were higher than that by the other methods under two conditions, which meant that the proposed algorithm performed better than SOBI-SWT and SSA.

**TABLE 3:** The average CRC values between removed EOG artifacts and EOG reference after EOG artifacts elimination by performing compared methods for 10 subjects.

	EICA	SOBI-SWT	SSA
Subject1	0.949	0.842	0.744
	0.957	0.922	0.805
Subject2	0.953	0.825	0.559
	0.960	0.928	0.721
Subject3	0.948	0.897	0.828
	0.950	0.937	0.740
Subject4	0.960	0.919	0.889
	0.970	0.942	0.572
Subject5	0.960	0.909	0.553
	0.957	0.885	0.598
Subject6	0.930	0.881	0.870
	0.957	0.922	0.966
Subject7	0.947	0.884	0.862
	0.964	0.940	0.846
Subject8	0.955	0.911	0.658
	0.955	0.928	0.798
Subject9	0.955	0.855	0.458
	0.953	0.878	0.444
Subject10	0.929	0.885	0.526
	0.925	0.878	0.690

*For each subject, the upper and lower rows correspond to values from resting state with eyes-open and emotional faces recognition task, respectively.*

## Discussion

It is an unavoidable fact that there exist numerous blink artifacts in the recorded EEG signals. The presence of EOG strongly obscures the quality of EEG signals, which increases the difficulty in further EEG analysis and can mislead to the interpretation of results. For this reason, it is necessary and important to design a method to decrease such artifacts in EEG recordings. In this paper, the EICA method, which combined EEMD and ICA, was proposed to automatically remove the EOG artifacts from multichannel EEG signals. First, the EOG-contaminated EEG signals were decomposed by ICA into multi-ICs. Then, the EOG-related ICs were automatically distinguished by kurtosis, while the other ICs were preserved. Next, EOG-related ICs were separated into a set of IMFs by using the EEMD method, and the EOG-related IMFs were automatically identified by correlation coefficient and rejected. Finally, the clean EEG signals without EOG artifacts were reconstructed by projecting IMFs and performing the inverse transform of ICA sequentially. The results of removing EOG artifacts from simulated EEG signals and real EEG signals both demonstrated that the proposed approach was capable of removing EOG artifacts and retaining the majority of EEG information.

In order to further investigate the effectiveness of the proposed EICA method, it was compared to other existing artifact rejection methods by using the same simulated datasets. Compared to these methods, the proposed method obtained the biggest increase in SNR and decrease in RMSE after EOG artifacts rejection, which indicated that EICA got the best performance of EOG artifacts elimination. Furthermore, the SOBI-SWT and SSA algorithms were employed to the real EEG data in comparison. The results demonstrated that the performance of the proposed method was also better than other two approaches in terms of CRC.

ADF based on regression approach (He et al., 2004; Wallstrom et al., 2004) was employed to eliminate EOG artifacts from mixed EEG signals. For the reconstructed signals (Supplementary Figure 1S), however, ADF seriously distorted the EEG signals. Meanwhile, the ADF method also lost much EEG information after the rejection of EOG artifacts (Supplementary Figure 1T). As we know, the ADF algorithm for artifact rejection is the process that adjusts weights constantly to obtain the optimal weights. Because of the non-deterministic and non-stationary characteristics of EEG signals, it is impossible to estimate such optimal weights. Consequently, relevant cerebral information can also be eliminated when removing EOG artifacts by ADF. Another class of artifact correction methods are based on BSS techniques such as ICA, SSA, and CCA (Iriarte et al., 2003; Flexer et al., 2005; Li et al., 2006; Gao et al., 2010; Zeng et al., 2013). In simulation, the corrected EEG signals by these BSS techniques indicated that EOG artifacts were removed from mixed EEG signals (Supplementary Figures 1M,O,Q). It is to be note that the artifact-related components decomposed by BSS contained neural activity. Hence, artifactual components were directly rejected, which may result in the loss of EEG information (Supplementary Figures 1N,P,R). Instead, the proposed method served components related to artifacts of eye blink as the input of EEMD. Thus, neural activity leaked to artifact-related components were extracted and recovered when the EEMD was used to remove EOG artifacts. Therefore, the performace of the proposed EICA technique was better than the ADF and BSS methods for EOG artifacts removal. Furthermore, the simulated results also indicated that the BSS approaches removed EOG artifacts from raw EEG signals more effectively in contrast to the ADF method, which was consistent with a previous study (Hoffmann and Falkenstein, 2008).

In this study, the AWICA and SOBI-SWT algorithms based on wavelet transform were applied for comparison to verify the performance of the proposed EICA approach. For AWICA (Mammone et al., 2012), each channel EEG of the mixed signals was first decomposed by DWT into four basic EEG rhythms or wavelet components (WCs). Subsequently, artifact-related WCs were identified and disentangled into a number of wavelet ICs by performing ICA. Finally, the artifact-related wavelet ICs were eliminated. Although WCs associated with low frequency contained most information of EOG, it implied that artifacts cannot be completely extracted. Thus, the EOG artifacts cannot be effectively removed (Supplementary Figure 1K). For SOBI-SWT (Mowla et al., 2015), first, the mixed signals were divided into a number of sources by applying SOBI. Then, the sources recognized as EOG artifacts were carried out by wavelet decomposition technique. Thus, the level-dependent fixed form threshold method of wavelet transform was used to detect and eliminate EOG artifacts. Generally speaking, both the mother wavelet and decomposition levels need to be manually chosen for SWT, which has a significant effect on the results. Compared to most other methods, no visible EOG artifacts can be observed in the clean EEG signals corrected by SOBI-SWT (Supplementary Figure 1E). In contrast to the AWICA and SOBI-SWT methods, the EICA algorithm decomposed the original signals into multi-ICs by using ICA, and EOG-linked ICs included almost all EOG components. By EEMD, EOG-linked ICs were further disentangled into EOG-related IMFs and EEG-related IMFs. Only the EOG-related IMFs were eliminated, and hence, some desired EEG information was retained. By visually comparing the waveforms of Supplementary Figures 1D,F, we found that EOG contents in the clean EEG signals by EICA were less than that by SOBI-SWT. EEMD is superior to other signal decomposition techniques such as wavelet transform for two main reasons: first, the EEMD algorithm is a completely data-driven signal decomposition method without a predefined basis function, and second, the decomposed oscillatory modes from the raw signal can well reflect the local characteristics of the signal. Thus, the EEMD method is preferable to other methods for analyzing non-stationary and non-linear electrophysiological signals (Wu and Huang, 2009). Therefore, the ability of removing EOG artifacts of the EICA method was superior to the AWICA and SOBI-SWT approaches.

We also compared the EICA method against the IMEMD approach (Wang et al., 2016). For IMEMD, first, the original EEG signals were decomposed into multiple multivariate IMFs (MIMFs) by using MEMD method. Second, EOG-related signals were extracted by reconstructing the MIMFs linked to EOG artifacts. Third, ICA was used to decompose EOG-related signals into multiple ICs. Fourth, artifact-linked ICs were automatically recognized by kurtosis and eliminated. Finally, the clean EEG signals without blink artifacts were reconstructed by performing the inverse transform of ICA and MEMD in sequence. From the denoising results, the EICA approach can effectively eliminate blink artifacts from the mixed signals with higher SNRs, since the majority of useful EEG information was preserved with lower RMSEs compared to IMEMD. The main distinction between the proposed method and the IMEMD approach is the information extraction of EOG artifacts. It is explained by the fact that the latter selects EOG-related MIMFs based on the way of averaging correlation coefficients between each MIMF and EOG reference and the former chooses EOG-related ICs by kurtosis. Thus, EOG information contained in EOG-related MIMFs is not as complete as that in EOG-linked ICs. Supplementary Figure 1G also confirmed that there were visibly residual EOG artifacts contaminating the first few channels. Therefore, the EICA method obtained a better performance than the IMEMD approach for the removal of EOG artifacts.

Furthermore, the combination methods of the EEMD algorithm and a BSS technique have been used for artifact removal, which perform EEMD first then the BSS algorithm second. However, both the EEMD-CCA (Chen et al., 2019) and EEMD-ICA (Mijovic et al., 2010) methods had a poor performance in comparison with the proposed method. Since the EOG-contaminated EEG signals were decomposed into a set of IMFs by EEMD, EOG-linked components were contained in multiple IMFs. It is difficult to ensure that the BSS algorithm was able to separate EOG artifacts from the IMFs unless there was a mutually linear relationship between the original sources and the IMFs (Mijovic et al., 2010). Even if BSS decomposed the IMFs into multiple components, EOG-linked components also contained much EEG information. Consequently, the rejection of artifactual components can lead to the loss of EEG information (Supplementary Figure 1J). Another disadvantage of EEMD-CCA for EOG artifacts removal is that the EOG artifacts and EEG activity both had high autocorrelation values where estimated sources were very difficulty to be distinguished with respect to the autocorrelation values after using CCA. Due to relatively lower autocorrelation values of muscle signals, in fact, the CCA-based algorithms are more suitable for muscle artifacts removal than the BSS-based methods (De Clercq et al., 2006; Gao et al., 2010; Chen et al., 2019). In addition, the desired source in the original signal was successfully extracted by applying EEMD-ICA (Mijovic et al., 2010). An important reason is that the oscillation-type signal was modeled as a sinusoidal waveform in simulation, which can be extracted from the original signal by EEMD. Thus, it was no surprise that the EEMD-ICA method did not work well when using the real EOG signal for simulation.

## Limitation

Although both the vertical electrooculogram (VEOG) and horizontal electrooculogram (HEOG) signals were collected, only the VEOG signal was employed for the simulation study. Thus, a limitation of the study is that the study did not test the ability to remove HEOG artifacts.

## Conclusion

In this study, a novel EICA method combining EEMD and ICA was proposed to remove EOG artifacts from contaminated EEG signals. It was intended to overcome the deficiency of eliminating artifact components directly by selecting artifact-related ICs as the input of EEMD, further improving the performance of the artifacts removal. The effectiveness of the proposed method was both verified by simulated and real EEG data. The results of study of simulated signals demonstrated that the proposed approach could successfully suppress EOG artifacts from simulated EEG signals and mostly maintain the brain activity with little distortion. For study based on real EEG signals, the results displayed that the CRC values obtained by the proposed approach were the highest among the compared methods. Thus, it was concluded that the proposed approach could also eliminate the blink artifacts in real EEG signals very well. The strengths of the EICA method used for rejecting EOG artifacts lied in the complete extraction of EOG-linked ICs and the removal of EOG-related IMFs while preserving the useful EEG information to the greatest extent. Furthermore, the experimental results illustrated that the proposed method had the best performance compared to other existing approaches.

## Data Availability Statement

The raw data supporting the conclusions of this article will be made available by the authors, without undue reservation.

## Ethics Statement

The studies involving human participants were reviewed and approved by the local ethics committee of Xi’an Jiaotong University. The patients/participants provided their written informed consent to participate in this study.

## Author Contributions

C-LT took charge of analyzing EEG data and drafting the manuscript. Y-YZ took charge of experiment design and EEG recording. WW and Y-YL took charge of subjects recruitment and EEG recording. GW took charge of algorithm programming. JX took charge of the project research, experiment design, and manuscript preparation. All authors contributed to the article and approved the submitted version.

## Conflict of Interest

The authors declare that the research was conducted in the absence of any commercial or financial relationships that could be construed as a potential conflict of interest.

## Publisher’s Note

All claims expressed in this article are solely those of the authors and do not necessarily represent those of their affiliated organizations, or those of the publisher, the editors and the reviewers. Any product that may be evaluated in this article, or claim that may be made by its manufacturer, is not guaranteed or endorsed by the publisher.
