# Supplementary material for: A Novel Method Based on Combination of Independent Component Analysis and Ensemble Empirical Mode Decomposition for Removing Electrooculogram Artifacts From Multichannel Electroencephalogram Signals
Source: Front Neurosci. 2021 Oct 11;15:729403. doi: 10.3389/fnins.2021.729403 (PMC8542780; doi:10.3389/fnins.2021.729403)
Supplement: Supplementary file 1 [file Data_Sheet_1.docx]

**Supplementary Material**

A


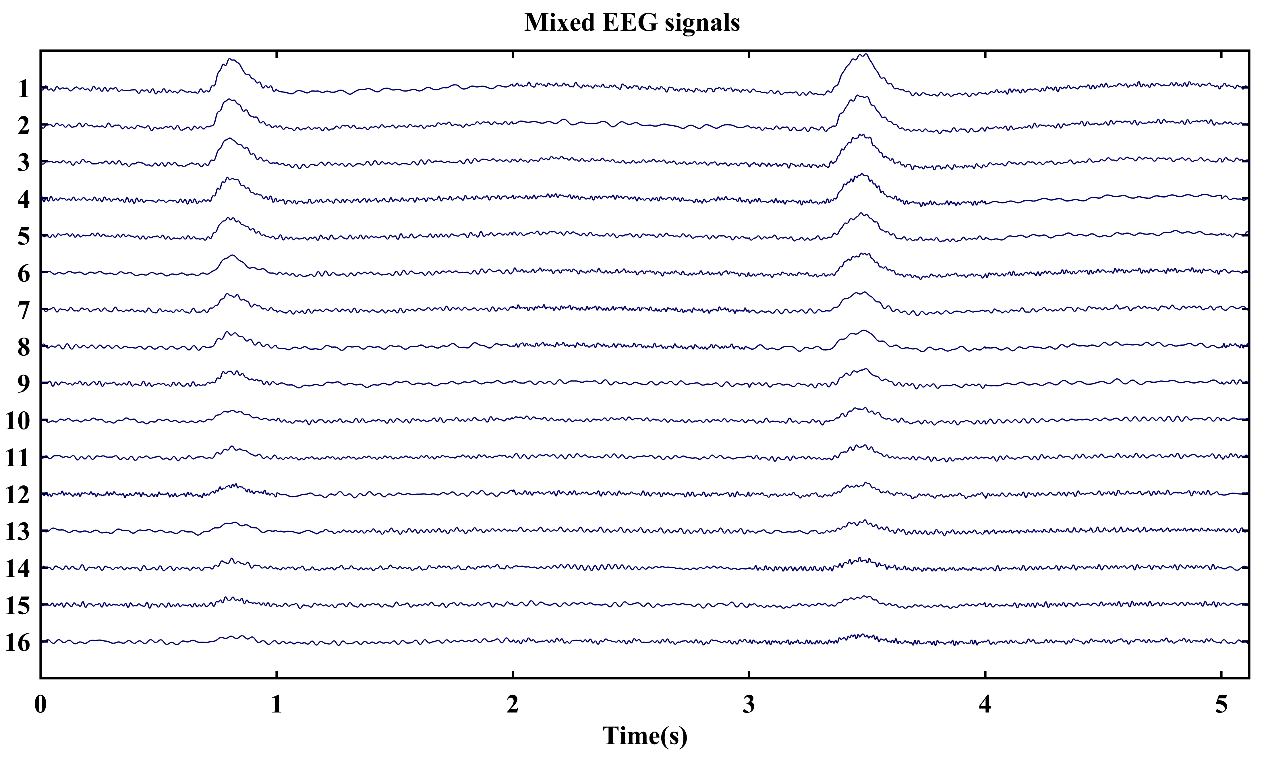


B


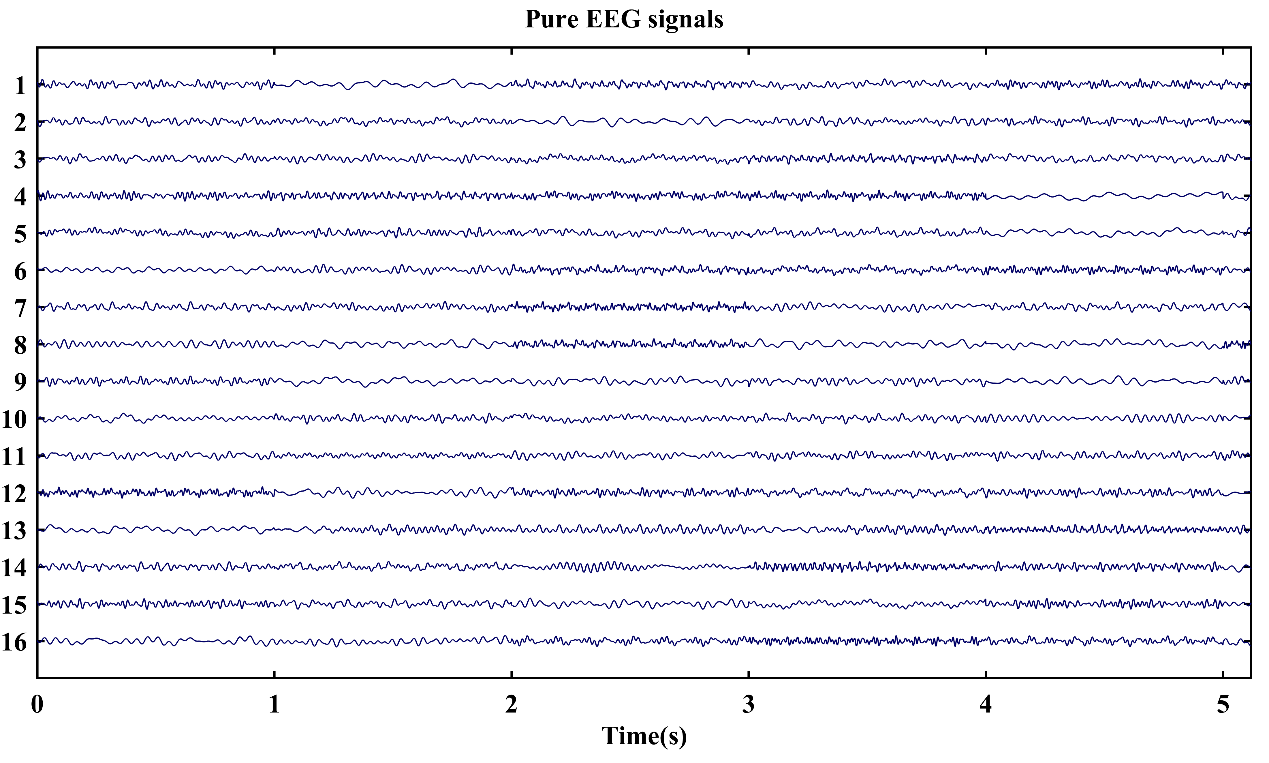


C


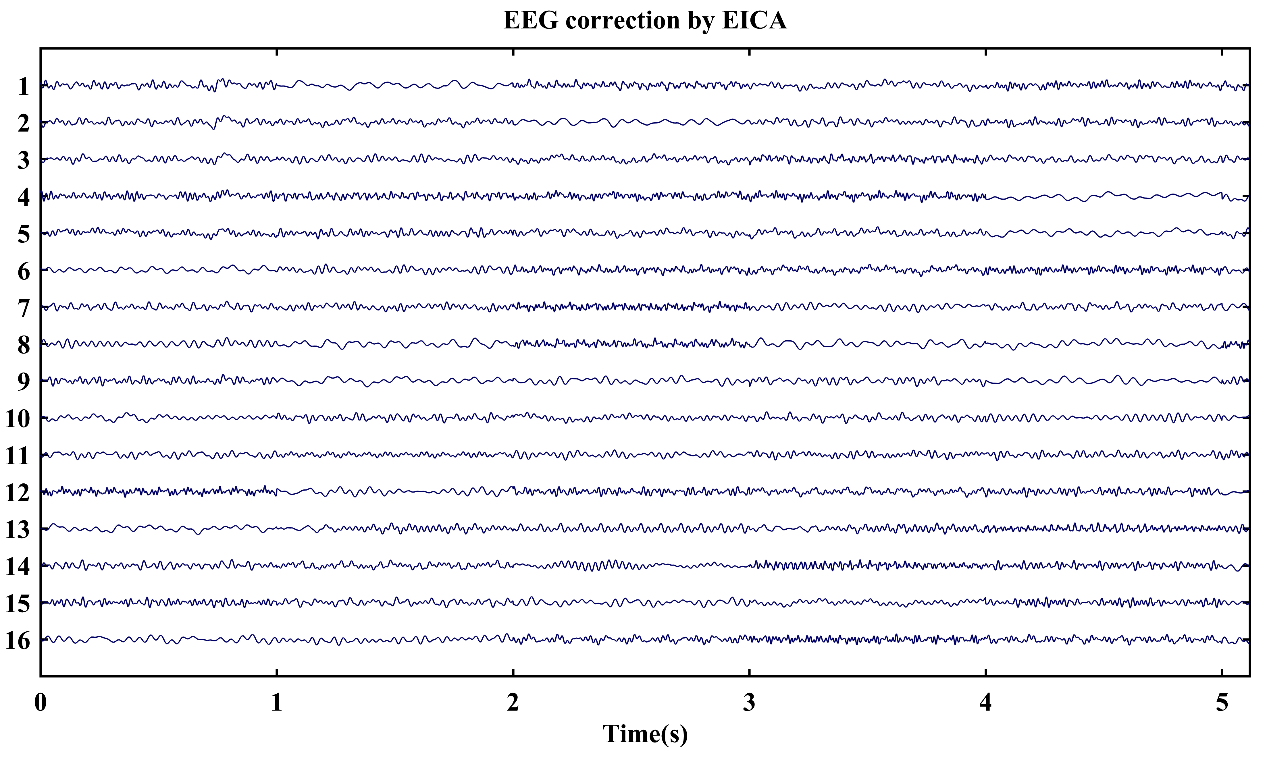


D


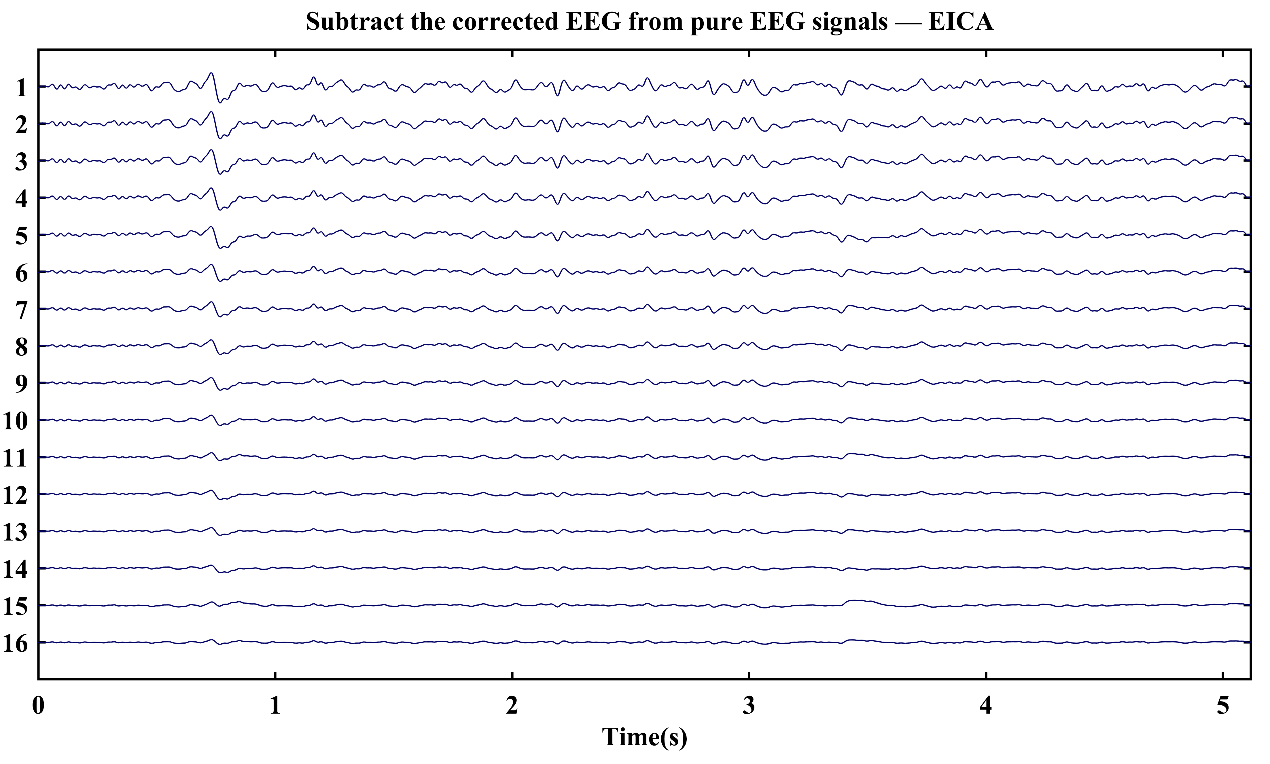


E


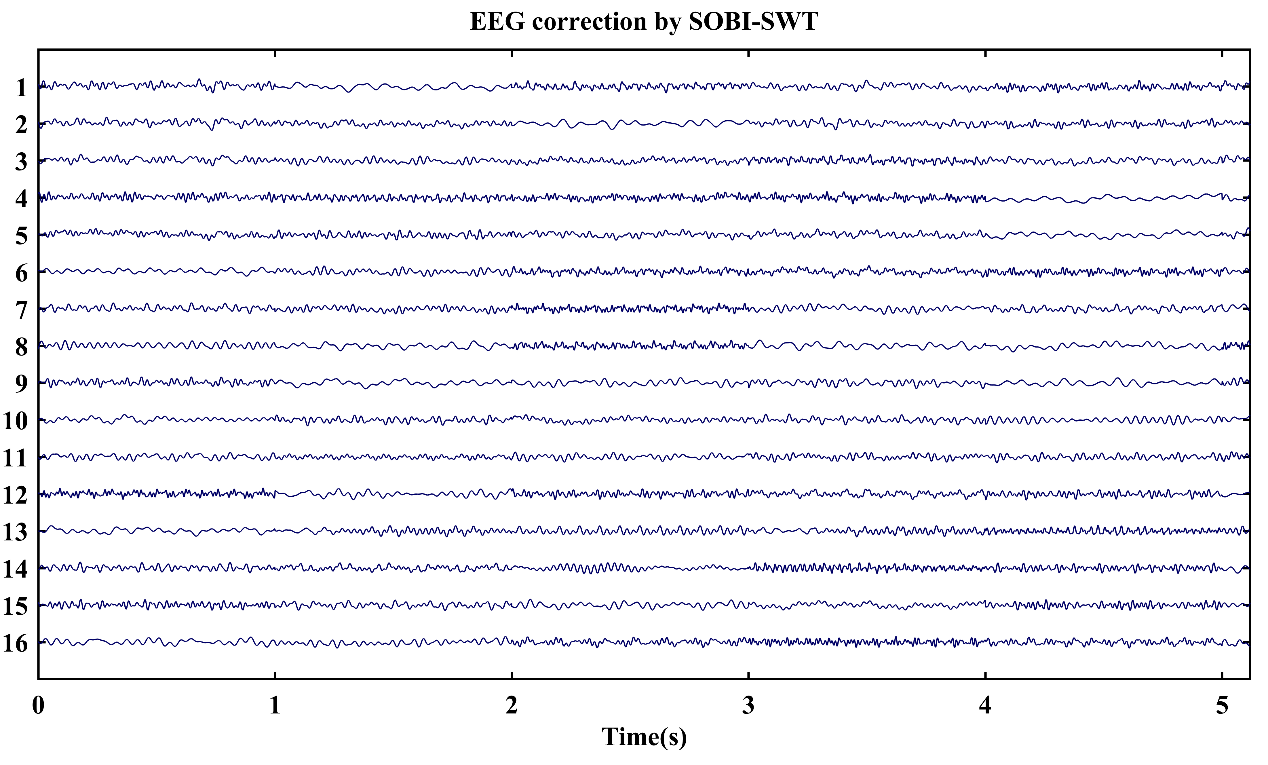


F


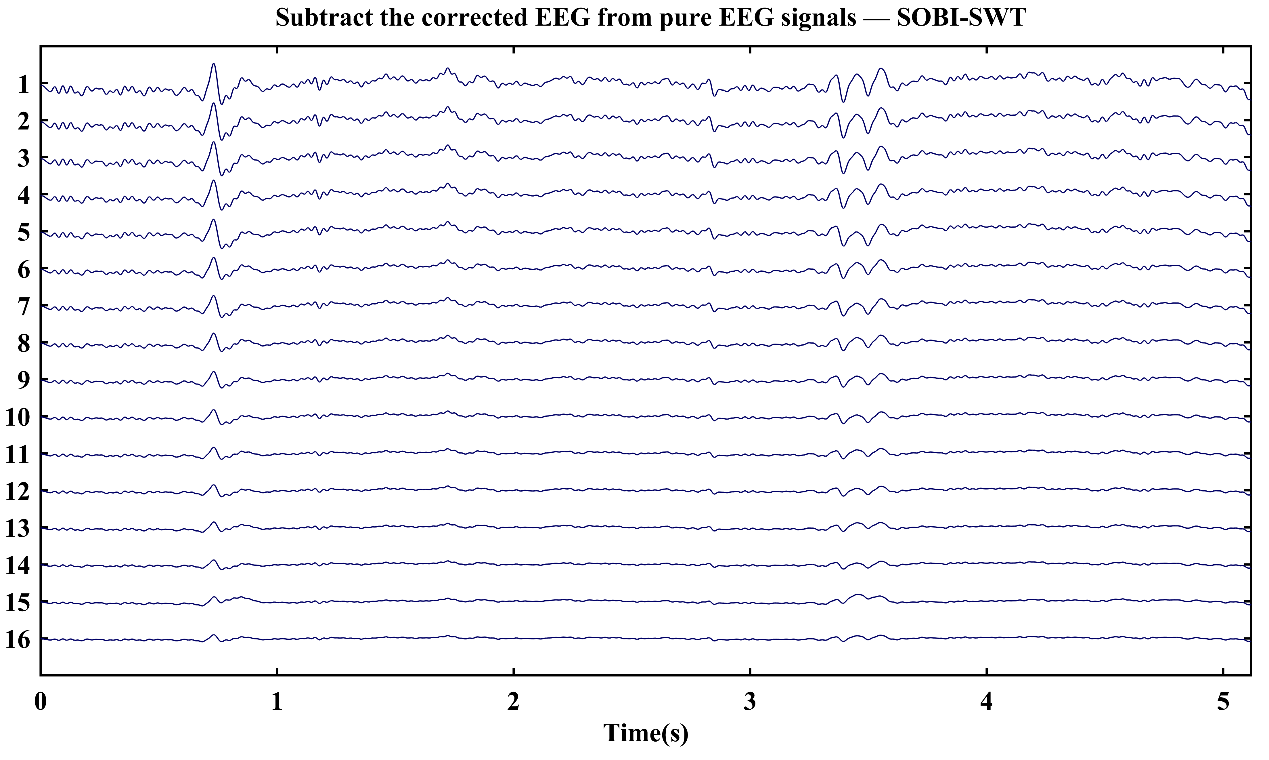


G


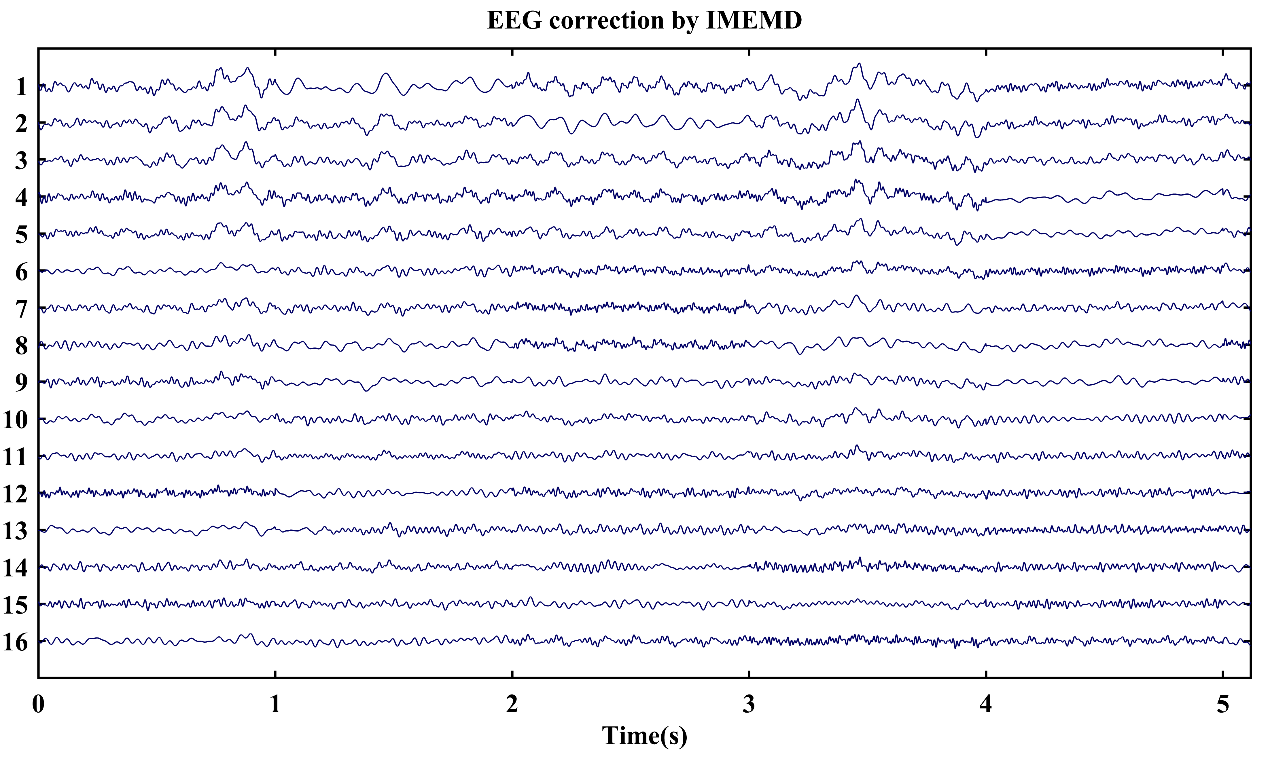


H


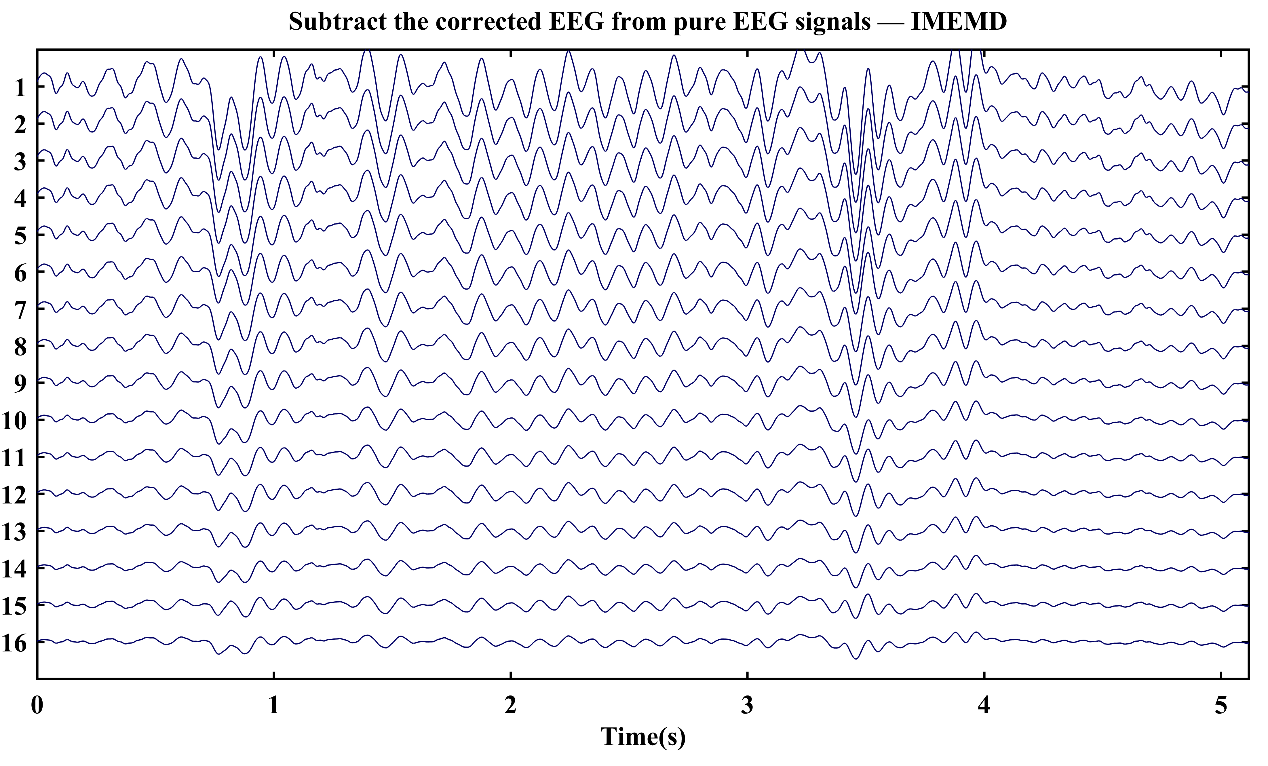


I


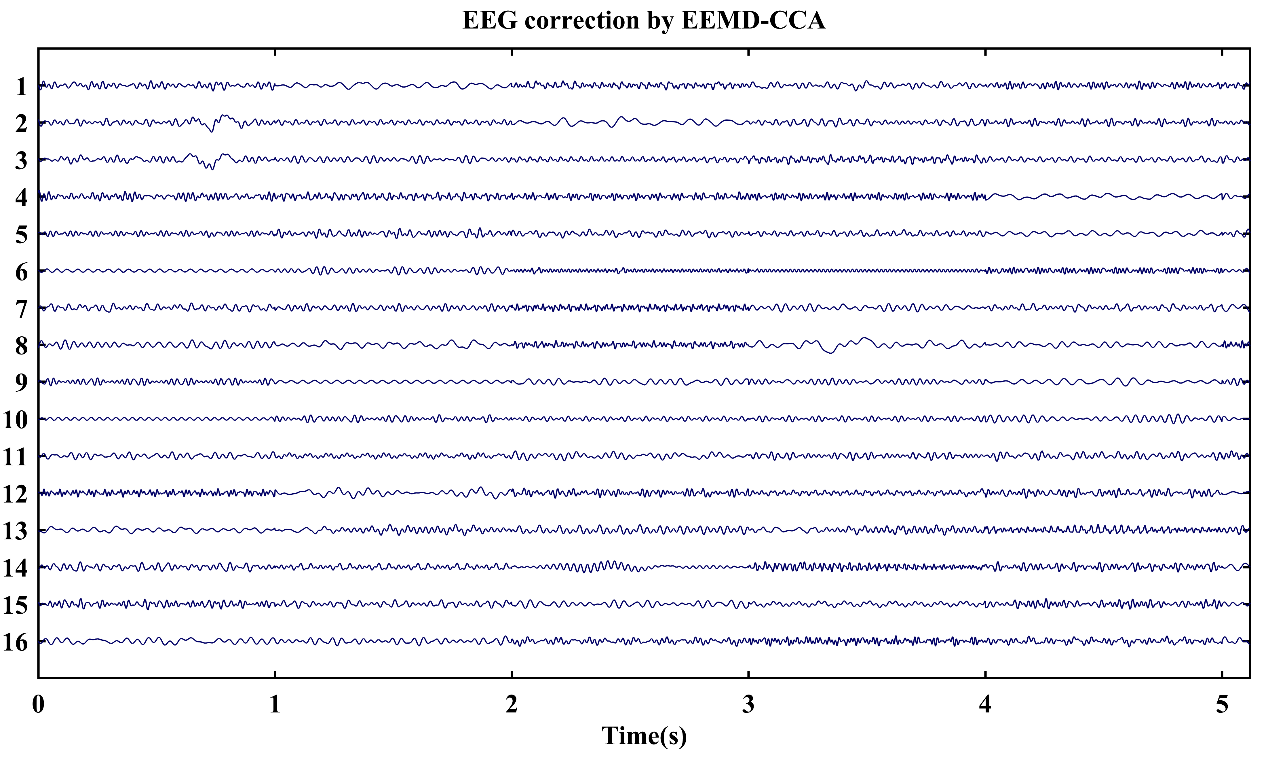


J


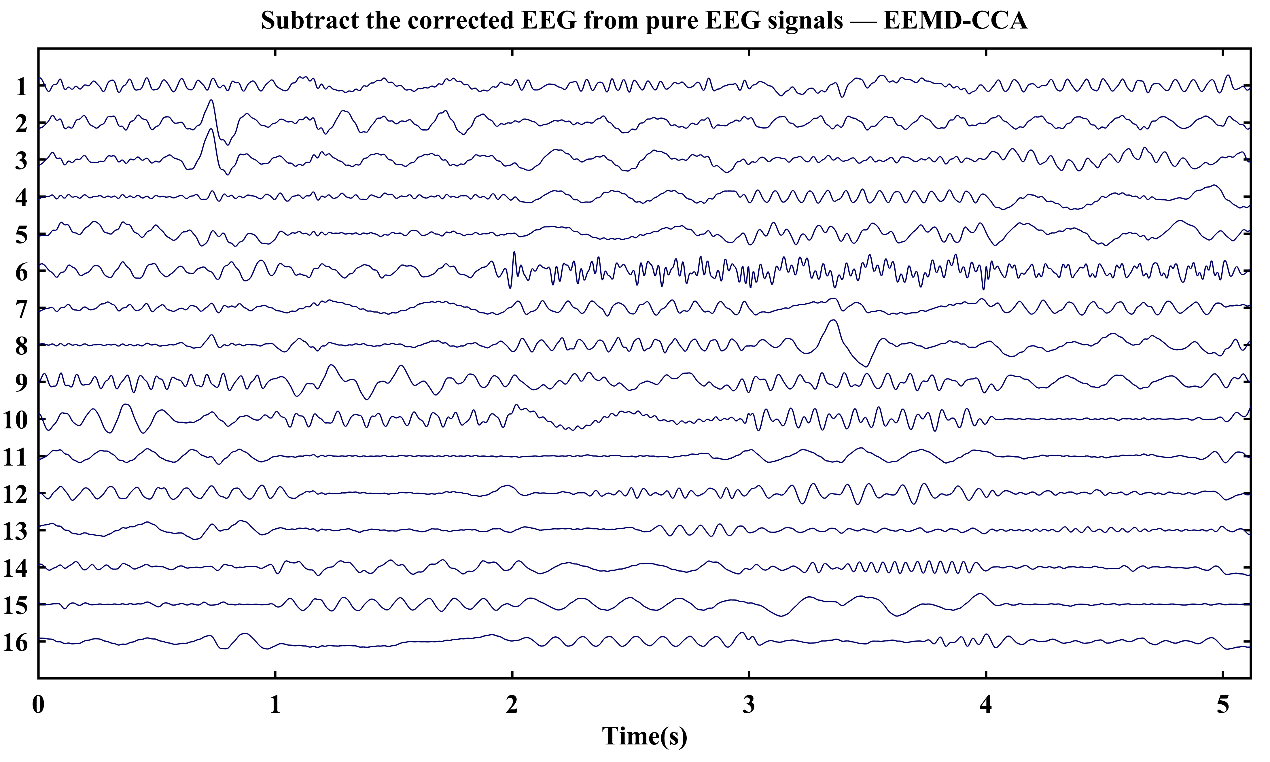


K


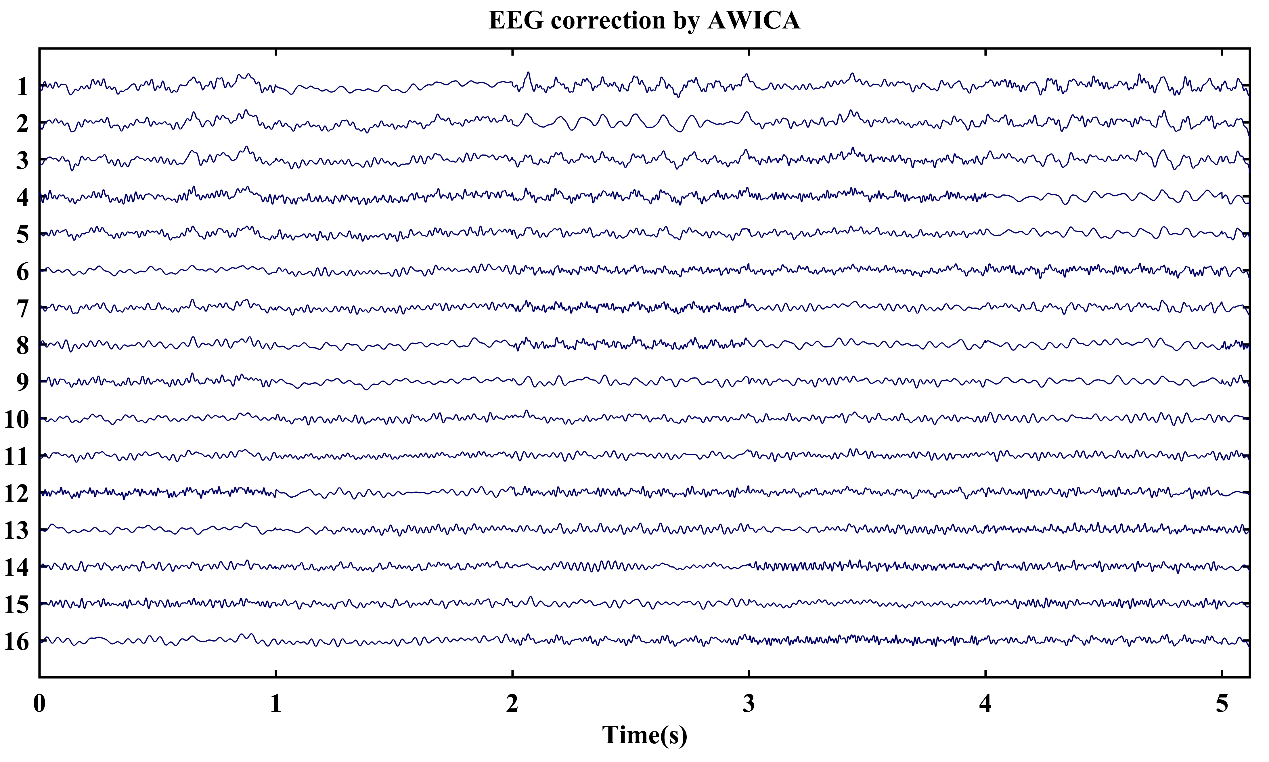


L


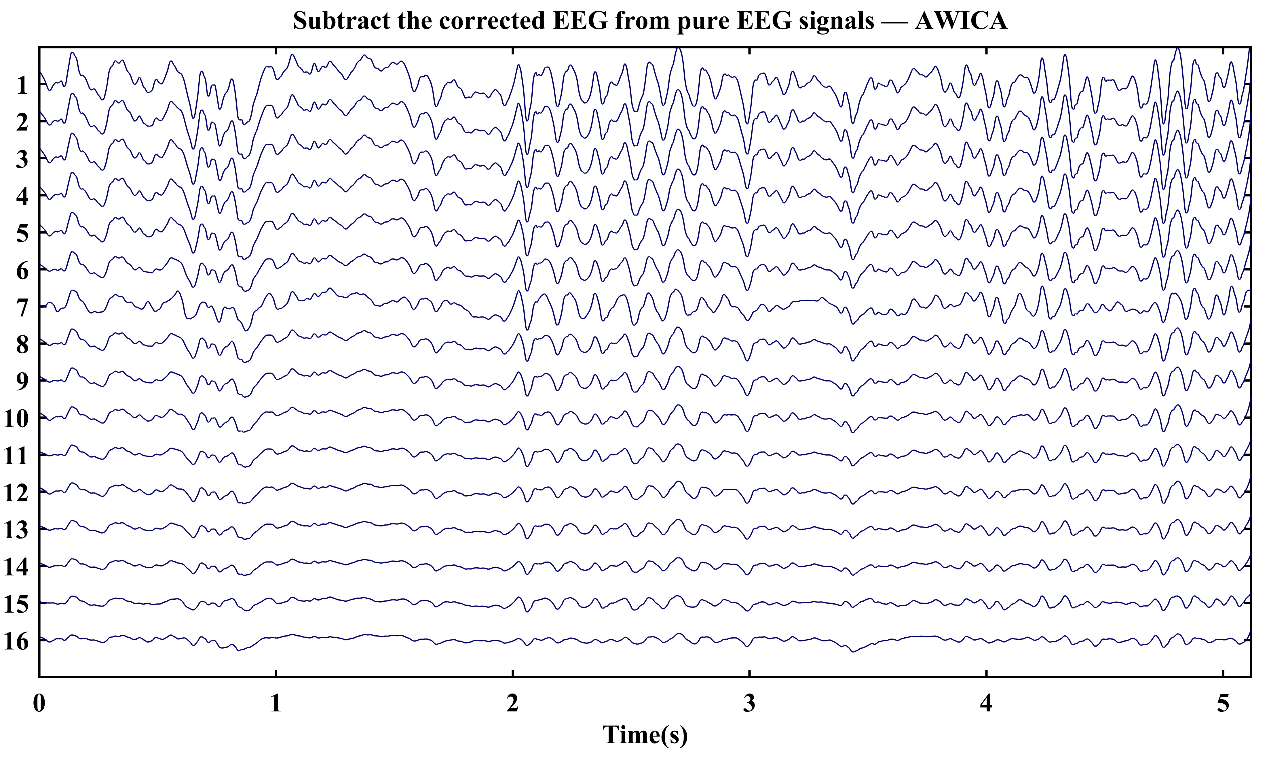


M


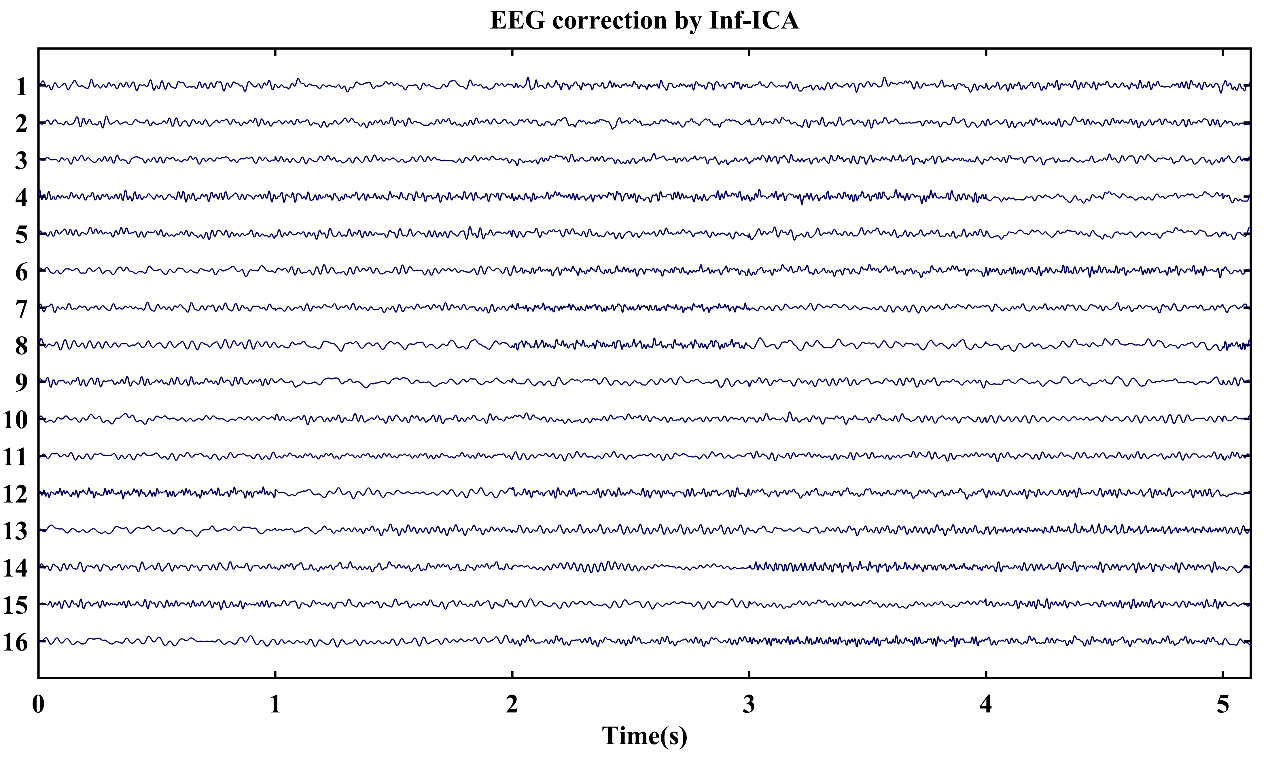


N


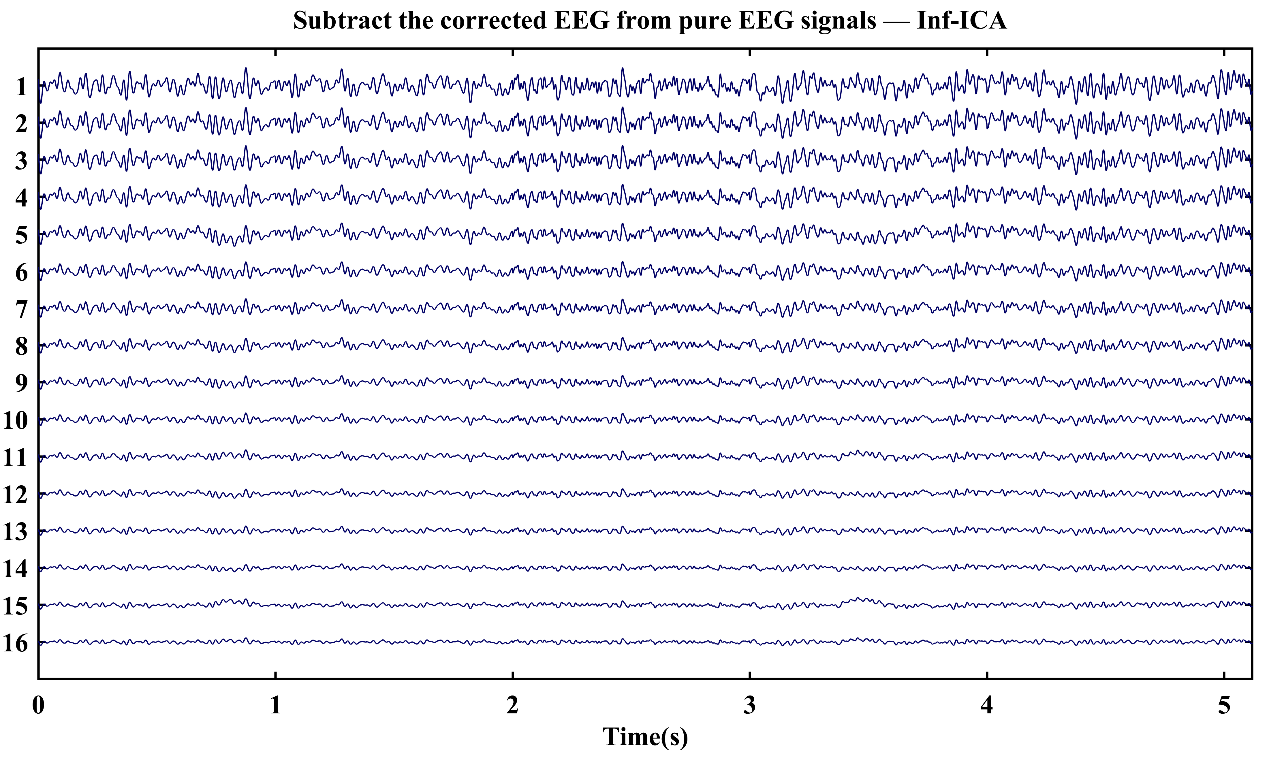


O


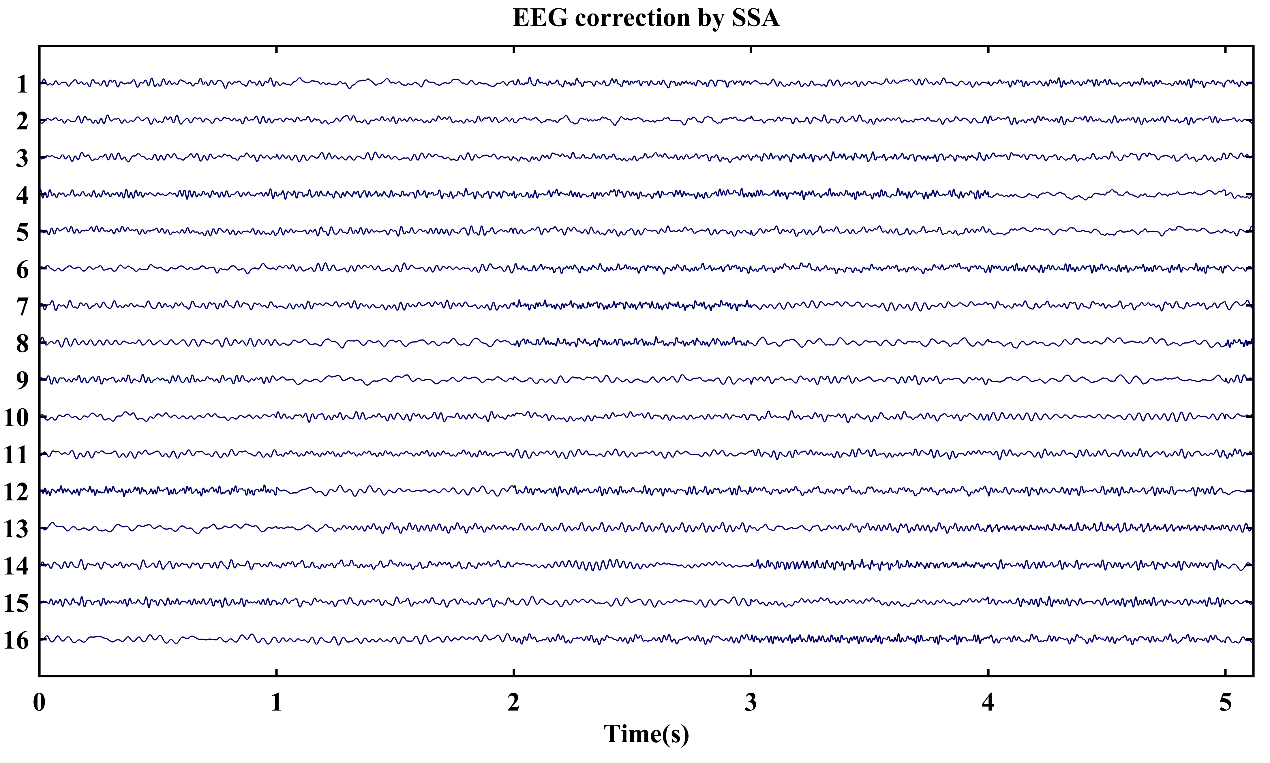


P


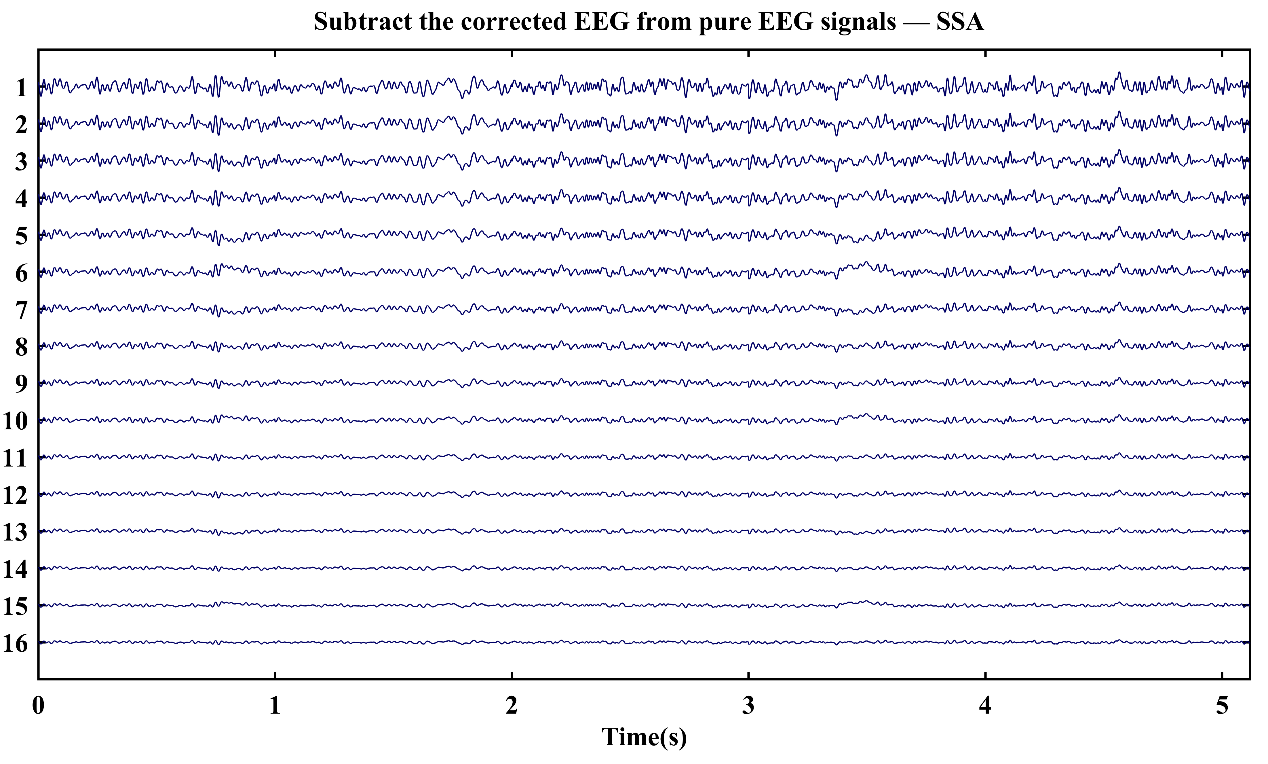


Q


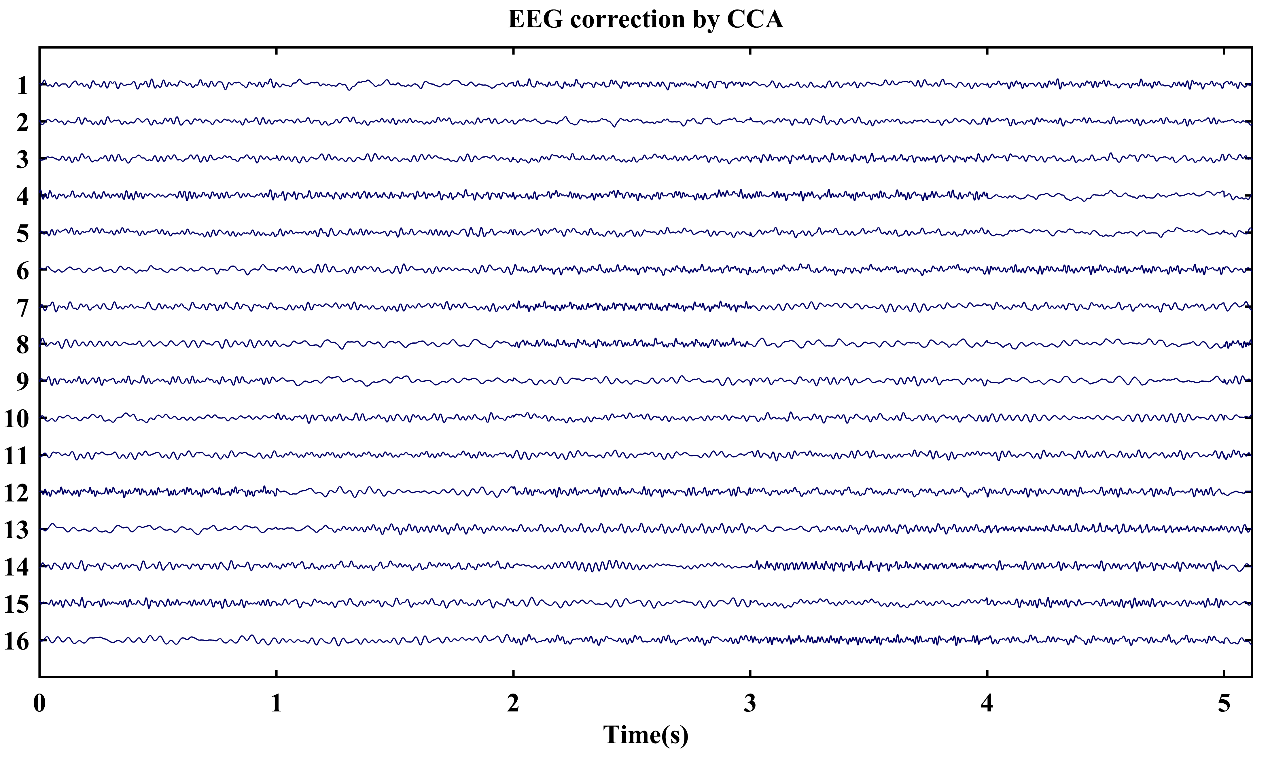


R


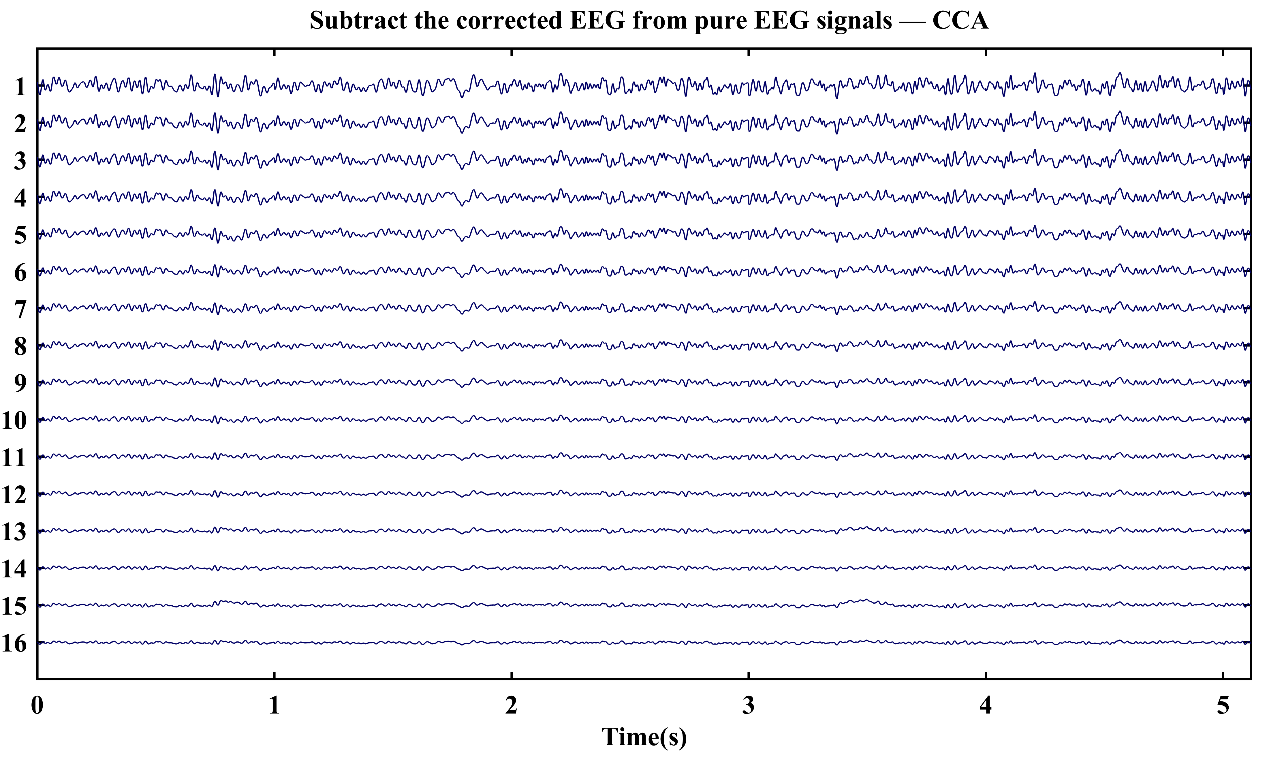


S


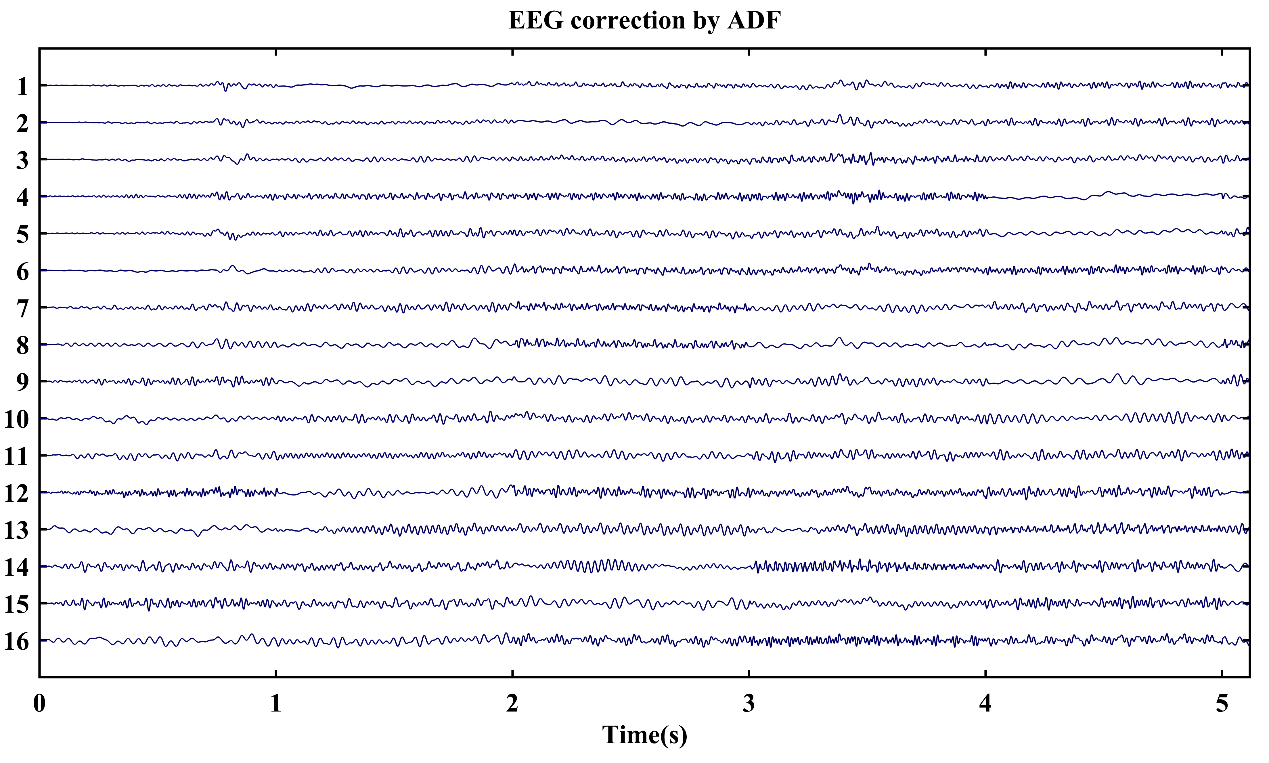


T


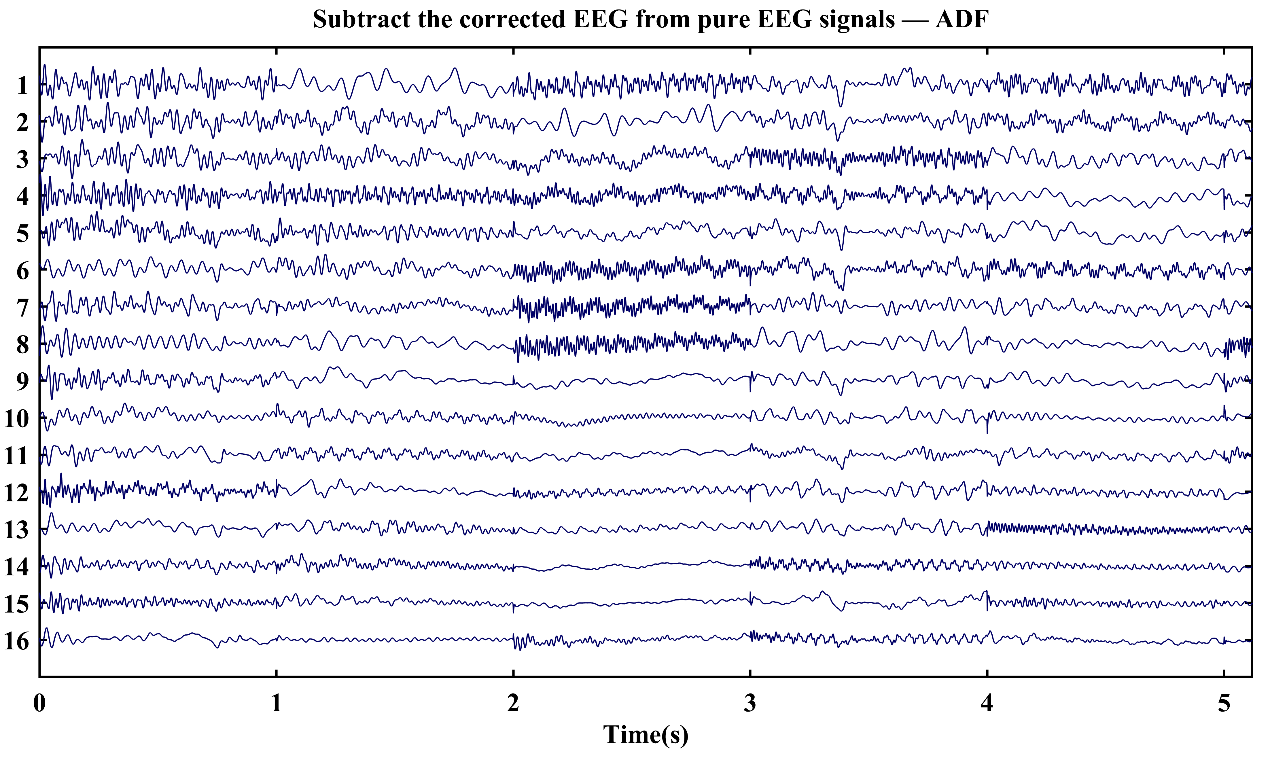


**FIGURE 1 | (A) Simulated mixed EEG signals, and (B) the pure EEG signals contained in the mixed data. The reconstructed EEG signals after artifacts removal by using different methods and the extracted signals obtained by subtracting the corrected EEG from pure EEG signals. (C) EEG correction by EICA, (D) the extracted signals—EICA, (E) EEG correction by SOBI-SWT, (F) the extracted signals—SOBI-SWT, (G) EEG correction by IMEMD, (H) the extracted signals—IMEMD, (I) EEG correction by EEMD-CCA, (J) the extracted signals—EEMD-CCA, (K) EEG correction by AWICA, (L) the extracted signals—AWICA, (M) EEG correction by Inf-ICA, (N) the extracted signals—Inf-ICA, (O) EEG correction by SSA, (P) the extracted signals—SSA, (Q) EEG correction by CCA, (R) the extracted signals—CCA, (S) EEG correction by ADF, and (T) the extracted signals—ADF.**
